# Metallodrug Profiling against SARS‐CoV‐2 Target Proteins Identifies Highly Potent Inhibitors of the S/ACE2 interaction and the Papain‐like Protease PL^pro^

**DOI:** 10.1002/chem.202103258

**Published:** 2021-11-23

**Authors:** Maria Gil‐Moles, Sebastian Türck, Uttara Basu, Andrea Pettenuzzo, Saurav Bhattacharya, Ananthu Rajan, Xiang Ma, Rolf Büssing, Jessica Wölker, Hilke Burmeister, Henrik Hoffmeister, Pia Schneeberg, Andre Prause, Petra Lippmann, Josephine Kusi‐Nimarko, Storm Hassell‐Hart, Andrew McGown, Daniel Guest, Yan Lin, Anna Notaro, Robin Vinck, Johannes Karges, Kevin Cariou, Kun Peng, Xue Qin, Xing Wang, Joanna Skiba, Łukasz Szczupak, Konrad Kowalski, Ulrich Schatzschneider, Catherine Hemmert, Heinz Gornitzka, Elena R. Milaeva, Alexey A. Nazarov, Gilles Gasser, John Spencer, Luca Ronconi, Ulrich Kortz, Jindrich Cinatl, Denisa Bojkova, Ingo Ott

**Affiliations:** ^1^ Institute of Medicinal and Pharmaceutical Chemistry Technische Universität Braunschweig Beethovenstr. 55 38106 Braunschweig Germany; ^2^ School of Chemistry National University of Ireland Galway University Road H91 TK33 Galway Ireland; ^3^ Department of Life Sciences and Chemistry Jacobs University Campus Ring 1 28759 Bremen Germany; ^4^ Department of Chemistry School of Life Sciences University of Sussex Falmer BN1 9QJ Brighton East Sussex UK; ^5^ Chimie ParisTech PSL University CNRS Institute of Chemistry for Life and Health Sciences Laboratory for Inorganic Chemical Biology 75005 Paris France; ^6^ Institut für Anorganische Chemie Julius-Maximilians-Universität Würzburg Am Hubland 97074 Würzburg Germany; ^7^ LCC–CNRS Université de Toulouse CNRS UPS Toulouse France; ^8^ Faculty of Chemistry Department of Organic Chemistry University of Łódź Tamka 12 91-403 Łódź Poland; ^9^ Department of Medicinal Chemistry and Fine Organic Synthesis Lomonosov Moscow State University Leninskie Gory 1/3 119991 Moscow Russia; ^10^ Institute of Medical Virology Universitätsklinikum Frankfurt Paul-Ehrlich-Str. 40 60596 Frankfurt Germany

**Keywords:** gold, metallodrugs, PL^pro^, polyoxometalates, SARS-CoV-2, silver, spike protein, titanocene

## Abstract

The global spread of the severe acute respiratory syndrome coronavirus 2 (SARS‐CoV‐2) has called for an urgent need for dedicated antiviral therapeutics. Metal complexes are commonly underrepresented in compound libraries that are used for screening in drug discovery campaigns, however, there is growing evidence for their role in medicinal chemistry. Based on previous results, we have selected more than 100 structurally diverse metal complexes for profiling as inhibitors of two relevant SARS‐CoV‐2 replication mechanisms, namely the interaction of the spike (S) protein with the ACE2 receptor and the papain‐like protease PL^pro^. In addition to many well‐established types of mononuclear experimental metallodrugs, the pool of compounds tested was extended to approved metal‐based therapeutics such as silver sulfadiazine and thiomersal, as well as polyoxometalates (POMs). Among the mononuclear metal complexes, only a small number of active inhibitors of the S/ACE2 interaction was identified, with titanocene dichloride as the only strong inhibitor. However, among the gold and silver containing complexes many turned out to be very potent inhibitors of PL^pro^ activity. Highly promising activity against both targets was noted for many POMs. Selected complexes were evaluated in antiviral SARS‐CoV‐2 assays confirming activity for gold complexes with N‐heterocyclic carbene (NHC) or dithiocarbamato ligands, a silver NHC complex, titanocene dichloride as well as a POM compound. These studies might provide starting points for the design of metal‐based SARS‐CoV‐2 antiviral agents.

## Graphical abstract

Despite their increasing relevance in medicinal chemistry, metal complexes are still underrepresented in compound screening libraries for drug discovery. In this work more than 100 metal complexes were evaluated as inhibitors of two targets in the SARS‐CoV‐2 life cycle, the interaction of the spike protein with the ACE2 receptor and the protease PL^pro^. The most active inhibitors were studied for antiviral effects in SARS‐CoV‐2 infected cells and led to the discovery of active compounds that will provide starting points for future drug design.

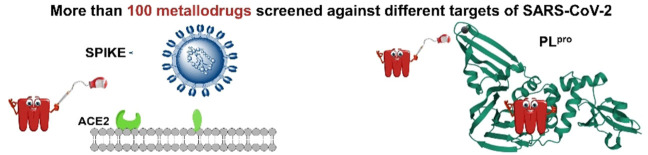

## Introduction

Started in late 2019, the global spread of the severe acute respiratory syndrome coronavirus 2 (SARS‐CoV‐2) during the year 2020 has caused an uncontrolled pandemic and an unprecedented global health crisis.^[1]^ While vaccines have become available within one year, there is still an immense need for effective therapeutic drugs. Vaccines are prophylactic but not therapeutic agents and can become less effective upon mutations of the virus. Moreover, the possible emergence of future strains of coronaviruses suggests that the discovery of novel antiviral agents is of utmost importance in terms of pandemic preparedness. Efforts in developing medicines for the coronavirus disease 2019 (Covid‐19) have been focusing on drug repurposing strategies with limited success. Inhibitors of viral protein targets, such as the polymerase inhibitors remdesivir or favipiravir, as well as immunomodulatory drugs directed at the host cell (e. g. dexamethasone), have been evaluated in clinical trials and now offer a very small and still insufficient pool of therapeutic options.^[2]^ Medicinal chemistry projects to design and develop novel inhibitors of SARS‐CoV‐2 target proteins have led to highly potent drug candidates, which might be translated into therapeutics to meet the urgent need for effective drugs.[^2^, ^3^] Very recently, Pfizer revealed the clinical drug candidate PF‐07321332^[4]^ and, during review of this manuscript, Merck has announced highly promising results of phase 3 clinical trials with the ribonucleoside analogue molnupiravir.^[5]^ We have recently reported on gold based drugs as effective inhibitors of SARS‐CoV‐2 protein targets.^[6]^ While the complexes only moderately inhibited the binding of the spike (S) protein to the angiotensin‐converting enzyme receptor 2 (ACE2), they displayed very promising activity against the SARS‐CoV‐2 papain‐like protease PL^pro^. Importantly, PL^pro^ inhibition correlated well with their capability to remove zinc from a zinc‐binding domain of the enzyme.

Antiviral effects have occasionally been reported for many types of metal‐based drugs, however, this area of therapeutic application has not been studied as intensively compared with for example anticancer metallodrug development. The topic of antiviral metal complexes has recently been reviewed comprehensively by Bergamini et al.^[7]^, confirming the impressive potential of metallodrugs in this area. In addition to gold complexes, also metallo‐supramolecular helicates^[8]^, bismuth^[9]^ and rhenium[^10^, ^11^] compounds have provided remarkable results against target proteins of SARS‐CoV‐2 in recent reports, palladium^[12]^ complexes have been suggested for further studies based on computational chemistry approaches, and zinc^[13]^ supplementation has been regarded as beneficial to Covid‐19 patients. The possible application of metal complexes against SARS‐CoV‐2 has been discussed and reviewed by Messori et al.[^14^, ^15^] and Karges and Cohen.^[11]^

In this paper, we report on the extensive profiling of >100 structurally diverse metallodrugs as potential inhibitors of the S/ACE2 interaction and/or PL^pro^ activity. Metal‐based compounds are usually underrepresented in drug screening libraries, which have a paucity of such compounds. The pool of investigated compounds includes several approved metal‐based therapeutics (e. g. aurothiomalate, silver sulfadiazine, thiomersal, cisplatin) together with a number of metal derivatives, which were provided by different research groups within the inorganic medicinal chemistry community.

### Selection of metallodrugs for the screening

**Figure 1:**
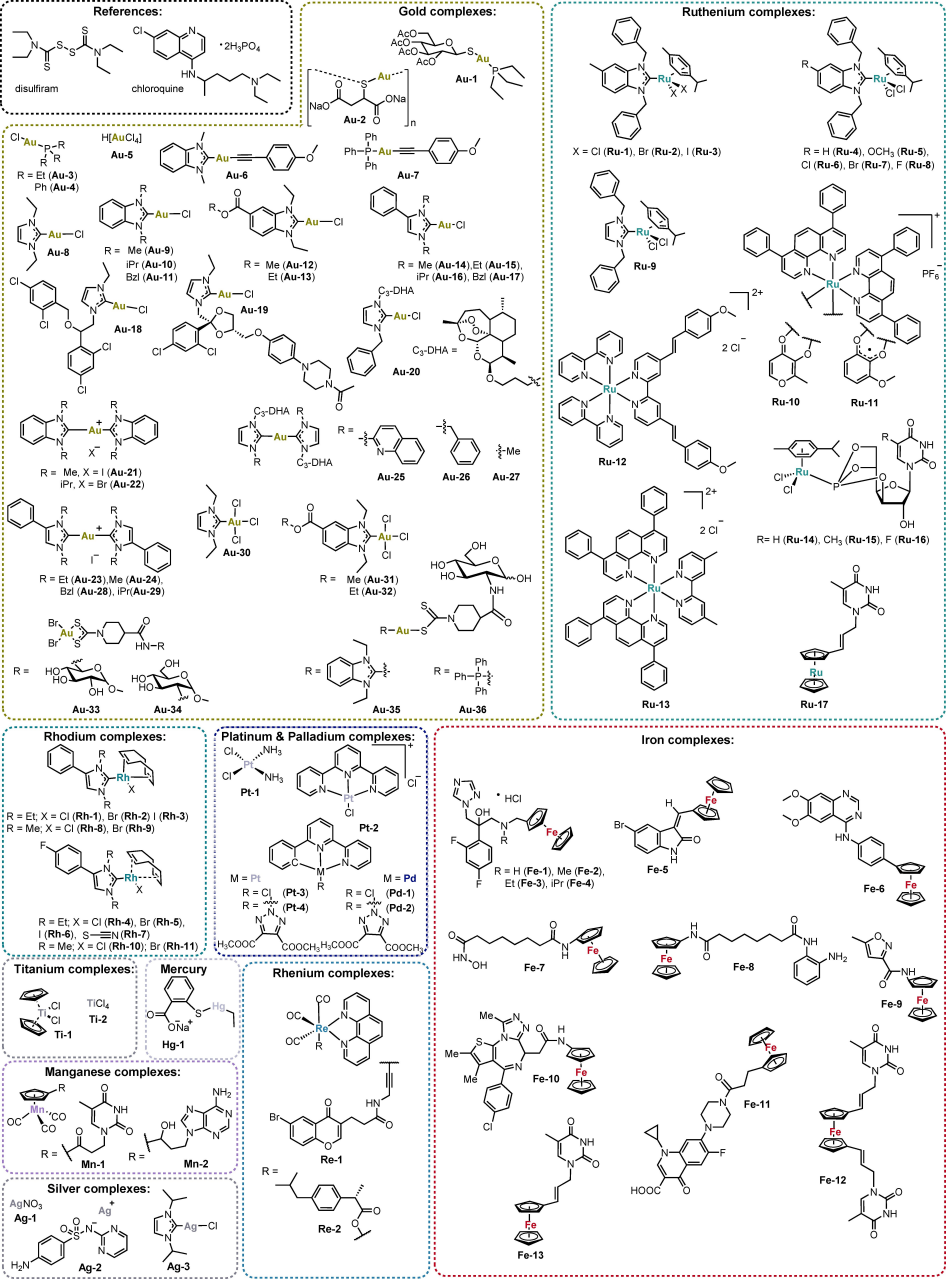
Mononuclear compounds investigated in this study.

**Figure 2:**
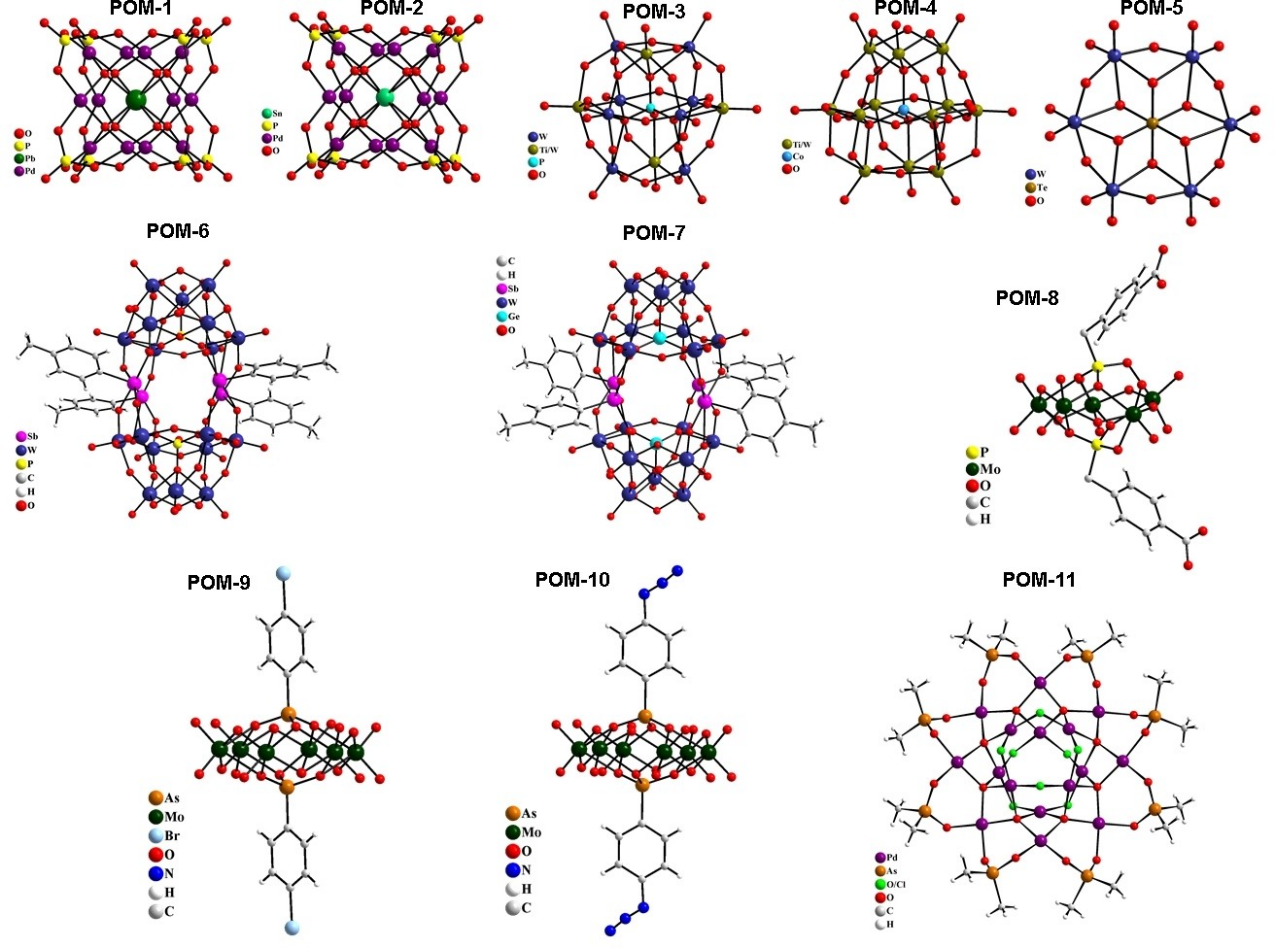
Polyoxometalates investigated in this study.

The pool of studied metal complexes includes 93 mononuclear compounds and 11 polyoxometalates (POMs). The group of mononuclear species contained 36 gold(I/III), 17 ruthenium(II/III), 13 iron(II/III), 11 rhodium(I), 4 platinum(II), 3 silver(I), 2 palladium(II), 2 titanium(IV), 2 rhenium(I), 2 manganese(II) and 1 mercury(II) complexes (see Figure 1). POMs included the following metal or metalloid elements: As(V), Co(II), Cs(I), Ge(IV), Mo(VI), Pb(II), Pd(II), Rb(I), Sn(IV), Te(IV), Ti(IV), and W(VI) (see Figure 2).

The potential antiviral application of various gold complexes had been reported before in the literature.^[7]^ Notably, for the lead compound of gold drugs, auranofin (**Au‐1**), antiviral activity against SARS‐CoV‐2 has already been confirmed within early drug repurposing efforts for Covid‐19 treatment.^[16]^ Our recent report demonstrated that inhibition of PL^pro^ and interference with the S protein are likely among the key mechanisms of the antiviral activity of auranofin (**Au‐1**) and other gold complexes.^[6]^ For the profiling, we selected an extended number of 36 structurally diverse gold complexes with gold in the oxidation states +1 or +3 and different types of co‐ligands, including N‐heterocyclic carbenes (NHCs), alkynyls, dithiocarbamates, phosphines and chlorides. Aurothiomalate (**Au‐2**) was also included as a reference approved gold drug.

While ruthenium complexes have been extensively studied as anticancer agents, there are very few reports on their antiviral activity.[^7^, ^17^] In this study, 17 ruthenium complexes were included with different types of ligands such as polypyridyls, *N*‐heterocyclic carbenes (NHC), phosphites and arenes. The chiral ruthenium complexes were all used as racemic mixtures.

In addition to its fundamental metabolic and biochemical roles, iron has been increasingly considered in metallodrug development. For example, the antimalarial ferroquine has reached late clinical trial stages.^[18]^ In this study, we screened 13 iron complexes, which like ferroquine, contain a ferrocene partial structure that is integrated into or attached to a bioactive scaffold (e. g. **Fe1**–**Fe4** are derivatives of the antifungal drug fluconazole, **Fe12** and **Fe13** are nucleoside derivatives).

In recent years rhodium complexes have been increasingly considered for metallodrug design based on their tunable chemical and biological properties (e. g. as inhibitors of enzymes or of protein‐protein interactions).^[19]^ Few reports are available on antiviral activity of rhodium species.^[20]^ In this work we analyzed 11 rhodium complexes with NHC ligands representing a class of organometallic compounds, for which we had reported promising biological properties recently.^[21]^

Fewer than 5 examples of each silver (e. g. silver sulfadiazine, **Ag‐2**), platinum (e. g. cisplatin, **Pt‐1**), palladium, titanium (e. g. titanocene dichloride, **Ti‐1**), rhenium, manganese and mercury (thiomersal, **Hg‐1**) mononuclear complexes were included in the study. Of those, in particular the rhenium, silver, palladium and platinum species appeared very promising based on reported antiviral activities of such metal complexes.[^7^, ^10^]

Polyoxometalates (POMs) are metal‐oxide clusters exhibiting a broad variety of structures constituting various transition metal‐ions in their high oxidation states. POMs have shown promise as antitumoral, antiviral, and antibacterial agents making them possible next‐generation metallodrugs.[^22^, ^23^] In particular, the reported antiviral effects of different POMs are of interest here.[^24^, ^25^] The possible recognition of the coronavirus S protein by POMs has been suggested in a recent review.^[26]^ In total 11 POMs were included in this study, Na_12_[PbO_8_Pd_12_ (PO_4_)_8_] ⋅ 38H_2_O (**POM‐1**),^[27]^ Na_12_[SnO_8_Pd_12_(PO_4_)_8_] ⋅ 43H_2_O (**POM‐2**),^[27]^ K_7_[Ti_2_PW_10_O_40_] ⋅ 6H_2_O (**POM‐3**),^[28]^ K_6_H_2_[CoW_11_TiO_40_] ⋅ 12H_2_O (**POM‐4**),^[29]^ Na_6_[TeW_6_O_24_] ⋅ 22H_2_O (**POM‐5**),^[30]^ Rb_5.5_Na_4.5_[{(*p*‐tolyl)Sb}_4_(PW_9_O_34_)_2_] ⋅ 40H_2_O (**POM‐6**),^[31]^ Rb_6_Na_6_[{(*p*‐tolyl)Sb}_4_(GeW_9_ O_34_)_2_] ⋅ 40H_2_O (**POM‐7**),^[31]^ Cs_3_K[(HO_2_CC_6_H_4_CH_2_P)_2_Mo_5_O_21_] ⋅ 5H_2_O (**POM‐8**),^[32]^ ((NH_2_)_3_C)_4_[(BrC_6_H_4_As)_2_Mo_6_O_24_] ⋅ 2H_2_O (**POM‐9**),^[32]^ ((NH_2_)_3_C)_4_[(N_3_C_6_H_4_As)_2_Mo_6_O_24_] (**POM‐10**),^[32]^ and [Pd_16_Na_2_O_26_ (OH)_3_Cl_3_((CH_3_)_2_As)_8_] ⋅ 0.25Na(CH_3_)_2_AsO_2_ ⋅ 17H_2_O (**POM‐11**)^[33]^ (Figure 2).

## Results

### Metal complexes as inhibitors of the interaction between the S protein and the ACE2 receptor

The life cycle of SARS‐CoV‐2 starts with the entry of the virus into the host cell and is mediated by the S protein on the virus surface, which binds to the ACE2 receptor in the host cell membrane. This fact makes the S protein an important target for the development of new drugs against SARS‐CoV‐2 that can act as entry inhibitors.[^34^, ^35^, ^36^] The S1 subunit of the S protein contains a receptor binding domain (RBD), which binds with specific residues in the N‐terminal helix of ACE2 to allow for viral attachment. Notably, the RBD contains nine cysteine residues, of which eight form four disulphide bonds^[37]^, that could be potential binding partners for metal ions. In terms of the interactions between the S RBD and ACE2, it has been observed that polar interactions, hydrogen bonds, and salt bridges are important at the interface between the RBD and ACE2.[^37^, ^38^] Considering, any metal or ligand capable of interfering with RBD‐ACE2 recognition might destabilise the S/ACE2 interaction and thus prevent the cellular entry of SARS‐CoV‐2.

**Figure 3:**
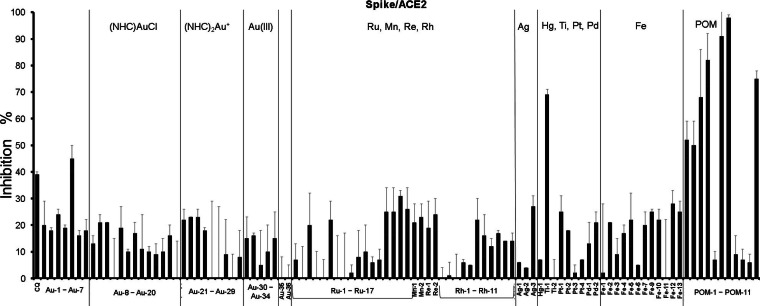
Inhibition of the S/ACE2 interaction by 20 μM of the test compounds; CQ: chloroquine.

For screening of the above described metallodrug library for inhibitors of the S/ACE2 interaction, an ELISA assay was used, which measures the interaction of the RBD of the SARS‐CoV‐2 S protein with the ACE2 receptor. In our previous report, gold metallodrugs had shown IC_50_ values in the 16–25 μM range in this assay. Accordingly, 20 μM concentrations of the test compounds were employed (Figure 3, Table S1).

The majority of the mononuclear metal complexes were found to be weakly active or inactive in these experiments. Titanocene dichloride (**Ti‐1**, 69 % inhibitory activity) was the only compound that reached >50 % inhibition of the S/ACE2 interaction. Additionally, low to moderate inhibition (25‐50 % inhibitory activity) was noted for **Au‐5**, **Ru‐14**–**17**, **Ag‐3**, cisplatin (**Pt‐1**), **Fe‐9**, **Fe‐12**, and **Fe‐13** as well as the reference drug chloroquine. Among those, the only gold species chloroauric acid (HAuCl_4_) **Au‐5** contains gold in the oxidation state +3. However, the other gold(III) complexes were poorly active or inactive. A common structural trend in the group of active compounds **Ru‐14–17**, **Fe12** and **Fe13** is that they contain ligands with nucleotide partial structures, while **Ag‐3** features a NHC ligand. By far, the most marked activity was obtained with the polynuclear POMs. In this group, seven derivatives displayed more than 50 % inhibition of the S/ACE2 interaction, while the remaining four derivatives (**POM‐5**, **POM‐8** to **POM‐10**) were virtually inactive showing less than 10 % inhibition.

Consequently, the mononuclear titanocene dichloride (**Ti‐1**) and the POMs **POM‐6**, **POM‐7** and **POM‐11** were selected for more detailed dose‐response studies. Very promising activity was observed for all four compounds with IC_50_ values in the low micromolar to submicromolar range (Table 1) thus by far exceeding the activity observed with several gold drugs in our recent report.^[6]^ With an IC_50_ value of 0.2 μM, **POM‐6** was the most active compound against the S/ACE2 interaction.

**Table 1:** IC_50_ values (±standard deviation) of selected complexes as inhibitors of the S/ACE2 interaction (n=2–3).

	IC_50_ (μM)
**Ti‐1** (titanocene dichloride)	3.9±0.3
**POM‐6**	0.2±0.1
**POM‐7**	0.3±0.1
**POM‐11**	2.5±0.3

### Metal complexes as inhibitors of the papain‐like protease PL^pro^

Proteases are well‐established targets in antiviral drug development. Selective inhibitors of viral proteases can interrupt the life cycle of the virus without affecting the host cells. Regarding SARS‐CoV‐2, in particular two viral cysteine proteases, the papain‐like protease (PL^pro^) and the 3‐chymotrypsin‐like protease (3CL^pro^, also called main protease M^pro^), are critically important for viral replication and thus represent attractive targets for inhibitor development.[^2^, ^39^, ^40^]

**Figure 4:**
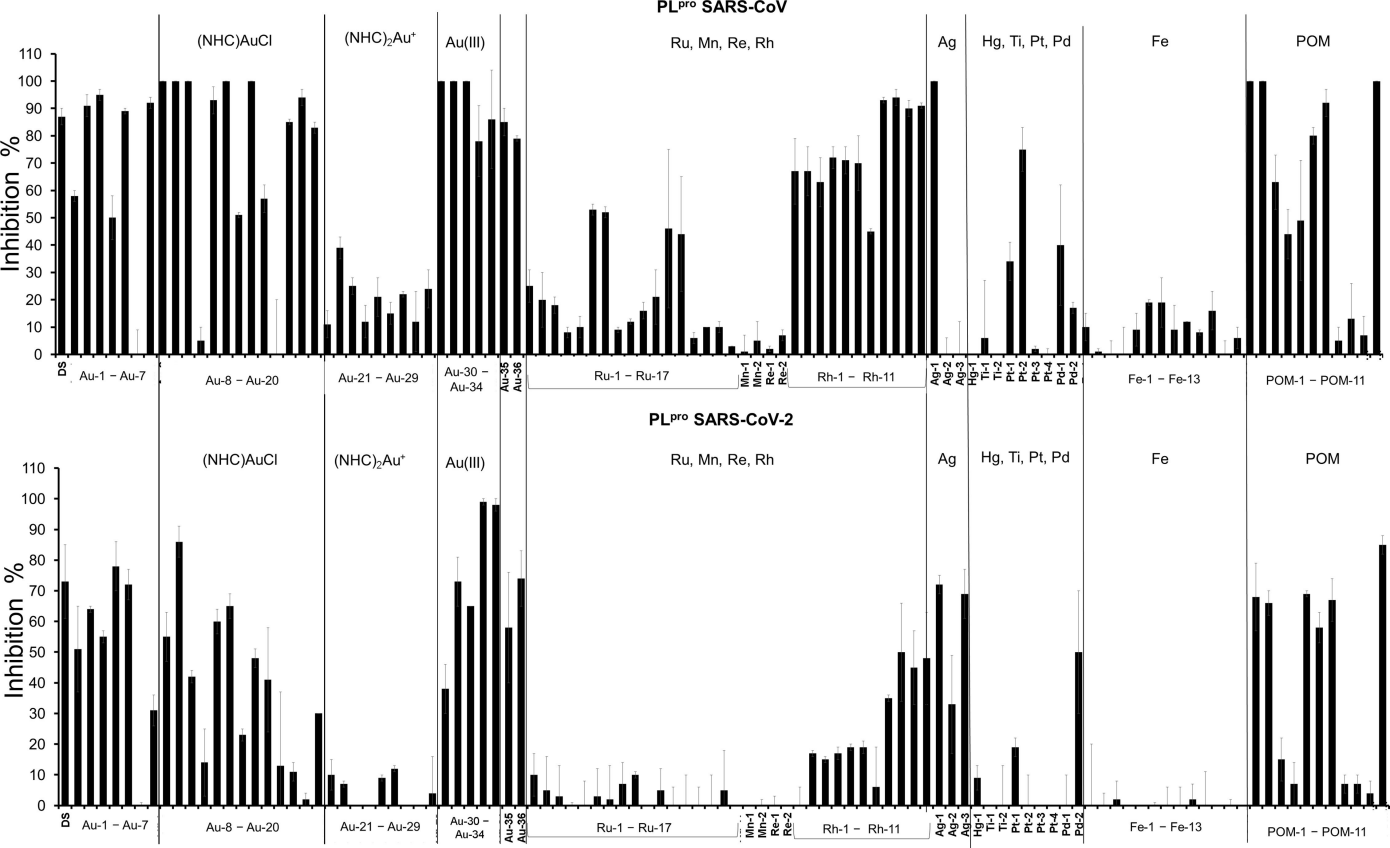
Inhibitory activity of 10 μM of the test compounds against SARS‐CoV PL^pro^ (top) and 1.0 μM of the test compounds against SARS‐CoV‐2 PL^pro^ (bottom); DS: disulfiram.

SARS‐CoV‐2 PL^pro^ shares 83 % sequence identity with the PL^pro^ of the coronavirus that had caused an earlier outbreak in 2003 (SARS‐CoV). The PL^pro^ enzymes from SARS‐CoV and SARS‐CoV‐2 feature several similar domains, of which the putative labile zinc‐binding domain and the catalytic cysteine cleavage domain contain conserved cysteine residues that can be targeted by thiol‐reactive probes.[^6^, ^39^, ^41^] In our previous report on gold complexes as inhibitors of PL^pro^ of SARS‐CoV and SARS‐CoV‐2, we observed stronger activity against the SARS‐CoV‐2 enzyme for the majority of the studied compounds.^[6]^ In accordance with these results, an inhibitor concentration of 10 μM was chosen for the assays with SARS‐CoV PL^pro^, while 1.0 μM was used for SARS‐CoV‐2 PL^pro^. The activities of both enzymes can be measured by a FRET based enzymatic assay (Figure 4, Tables S2 and S3).

#### Gold complexes as inhibitors of coronavirus PL^pro^

The overall most striking result was the high activity of many gold complexes against both types of PL^pro^. The gold reference compounds (auranofin (**Au‐1**), aurothiomalate (**Au‐2**), (triethylphosphine)AuCl (**Au‐3**), (triphenylphosphine)AuCl (**Au‐4**), HAuCl_4_ (**Au‐5**)) all led to >50 % of enzyme inhibition at the chosen concentrations.

With a few exceptions (**Au‐11** and **Au‐17**), which were inactive against both types of PL^pro^, all complexes of the type (NHC)AuCl were active against at least one type of the protease. Among those, **Au‐8**, **Au‐9**, **Au‐12** and **Au‐13** triggered more than 50 % inhibition of both enzymes. The two inactive complexes, **Au‐11** and **Au‐17**, both carry bulky benzyl groups at the NHC nitrogen atoms. In general, larger substituents on this position appeared to have a negative effect in particular for inhibition of SARS‐CoV‐2 PL^pro^. Regarding the (NHC)AuCl conjugates with bioactive ligands, **Au‐18**, **Au‐19** and **Au‐20**, high activities were noted against SARS‐CoV PL^pro^ (>80 % inhibition at 10 μM), but only the artemisinine derivative **Au‐20** was an at least moderate inhibitor of SARS‐CoV‐2 PL^pro^ (30 % inhibition at 1.0 μM).

In contrast to these generally strong activities with (NHC)AuCl complexes, neither the nine tested complexes of the type Au(NHC)_2_^+^ nor the (alkynyl)Au(I)(NHC) **Au‐6** reached >50 % enzyme inhibition at the chosen concentrations, which confirms our previous results with complexes of this type.^[6]^

The (alkynyl)Au(I)(phosphine) complex **Au‐7** and the (NHC)AuCl_3_ complexes **Au‐30**, **Au‐31** and **Au‐32** were good inhibitors of both types of PL^pro^.

Very strong inhibition of both types of PL^pro^ was recorded for the glycoconjugates **Au‐33** to **Au‐36**. Among those, the gold(III) dithiocarbamates **Au‐33** and **Au‐34** were among the most active compounds of this screening study.

Based on the above described results, ten of the most active gold complexes were selected for determination of IC_50_ values against SARS‐CoV and SARS‐CoV‐2 PL^pro^ (Table 2). Against PL^pro^ of SARS‐CoV values in the range of 0.3 to 1.2 μM and against SARS‐CoV‐2 PL^pro^ values in the range of 0.1 to 1.5 μM were obtained, confirming the high potential of gold compounds as protease‐inhibiting antiviral drugs. Submicromolar IC_50_ values against both enzymes were found with aurothiomalate (**Au‐2**), **Au‐31**, **Au‐35**, and **Au‐36**. For the gold(III) dithiocarbamates **Au‐33** and **Au‐34**, which were the most active SARS‐CoV‐2 PL^pro^ inhibitors identified in this study, a strong preference for the enzyme from SARS‐CoV‐2 was observed, while **Au‐9** and **Au‐12** were stronger inhibitors of the SARS‐CoV enzyme.

**Table 2:** IC_50_ values (±standard deviation) of selected metal complexes against PL^pro^ from SARS‐CoV and SARS‐CoV‐2 (n=2–3).

	SARS‐CoV PL^pro^ (μM)	SARS‐CoV‐2 PL^pro^ (μM)
**Au‐2** (aurothiomalate)	0.69±0.20	0.60±0.25
**Au‐3**	1.20±0.44	1.10±0.12
**Au‐8**	2.3±0.5	1.4±0.16
**Au‐9**	0.35±0.11	1.46±0.44
**Au‐12**	0.33±0.11	1.10±0.06
**Au‐31**	0.49±0.32	0.99±0.08
**Au‐33**	1.04±0.27	0.21±0.04
**Au‐34**	1.15±0.34	0.09±0.04
**Au‐35**	0.71±0.05	0.41±0.14
**Au‐36**	0.33±0.16	0.14±0.09
**Rh‐9**	6.7±2.4	2.2±1.0
**Ag‐1**	12±2	0.45±0.21
**Ag‐2** (silver sulfadiazine)	29±7	1.03±0.04
**Ag‐3**	11.4±0.05	0.18±0.07
**POM‐1**	0.95±0.06	0.17±0.11
**POM‐2**	0.82±0.17	0.54±0.08
**POM‐5**	19.3±4.6	5.8±1.8
**POM‐6**	9.1±1.6	0.20±0.03
**POM‐7**	2.4±1.6	0.46±0.19
**POM‐11**	0.71±0.2	0.13±0.04

#### Other mononuclear metal complexes as inhibitors of PL^pro^

The screening process revealed very low or negligible activity for all complexes containing iron, manganese, mercury, rhenium, and titanium. Regarding complexes with palladium, platinum and ruthenium, some examples with promising activities could be identified.

Among the group 10 metals, **Pt‐2** was the most active species towards SARS‐CoV PL^pro^ with 75 % inhibition at 10 μM and **Pd‐2** registered a 50 % inhibition of SARS‐CoV‐2 PL^pro^ at 1.0 μM, which was the highest activity against the enzyme from SARS‐CoV‐2 for this group of complexes.

With three complexes of the type (*p*‐cymene)(NHC)Ru(II)Cl_2_, **Ru‐1**, **Ru‐6** and **Ru‐7**, and the two polypyridyl species **Ru‐12** and **Ru‐13**, 5 out of the 17 investigated ruthenium compounds showed activity against PL^pro^ from SARS‐CoV (25–53 % inhibition at 10 μM), which might provide a starting point for further drug development. Concerning the polypyridyl complexes, the double positive charge of **Ru‐12** and **Ru‐13** appears to be beneficial, as the monocationic species **Ru‐10** and **Ru‐11** were significantly less active.

In contrast, some square‐planar rhodium(I) complexes of the general formula [RhX(cod)(NHC)] (with X=halide, cod=1,5‐cyclooctadiene, and NHC=N‐heterocyclic carbene) were also identified as good inhibitors of both PL^pro^ enzymes. There was a clear preference for N‐methyl substituents vs. N‐ethyl groups at the NHC ligand. The most active derivative **Rh‐9** was selected for IC_50_ determination and was found to inhibit both enzymes in the low micromolar range (Table 2).

While silver nitrate (**Ag‐1**) completely inhibited SARS‐CoV PL^pro^ activity at 10 μM, the other investigated silver compounds, silver sulfadiazine (**Ag‐2**) and **Ag‐3**, in which the metal is more firmly coordinated (e. g. by a NHC ligand), were inactive against this enzyme. In contrast, all silver compounds were good inhibitors of SARS‐CoV‐2 PL^pro^ at 1.0 μM. All three silver species were selected and their IC_50_ values determined. Notably, with values in the range of 0.2 to 1.0 μM, the silver complexes had comparable activity with the most active gold metallodrugs against SARS‐CoV‐2 PL^pro^ (Table 2). Substantially lower activity (IC_50_ values in the range of 11 to 29 μM) was noted against SARS‐CoV PL^pro^, confirming the selectivity for the SARS‐CoV‐2 enzyme observed in the single dosage screening.

#### Polyoxometalates as inhibitors of PL^pro^

Most of the POMs showed very good activity as inhibitors of PL^pro^. Against SARS‐CoV PL^pro^, all the POMs, except **POM‐8** to **POM‐10**, showed good activity at 10 μM, whereas against the enzyme from SARS‐CoV‐2, six out of the eleven studied compounds displayed strong effects at 1.0 μM. **POM‐4** was active only against the enzyme from SARS‐CoV, while **POM‐8** to **POM‐10** were inactive against both enzymes. The six most active compounds were selected for determination of their IC_50_ values (Table 2).

All six POMs displayed higher activity against the enzyme from SARS‐CoV‐2 than against that from SARS‐CoV. Against PL^pro^ SARS‐CoV, **POM‐1**, **POM‐2** and **POM‐11** reached IC_50_ values in the submicromolar concentration range, whereas **POM‐5** to **POM‐7** afforded low micromolar values. With the exception of **POM‐5** with an IC_50_ value of 5.8 μM, all compounds were highly active against PL^pro^ from SARS‐CoV‐2 (IC_50_ values in the range of 0.1 to 0.5 μM). The most active inhibitors of this enzyme were **POM‐1**, **POM‐6** and **POM‐11**.

### Antiviral effects against SARS‐CoV‐2

In order to select compounds for antiviral activity screening in SARS‐CoV‐2 infected cells, initially the toxicity of the complexes listed in Tables 1 and 2 was determined in Caco‐2 and CaLu‐3 cell lines (see Supporting Information). Regarding the mononuclear complexes, aurothiomalate (**Au‐2**), **Au‐33**, **Au‐34** and titanocene dichloride (**Ti‐1**) did not trigger significant cytotoxic effects up to the highest investigated concentration (500 μM), whereas the other mononuclear complexes turned out to be cytotoxic at different levels. As for the POMs, **POM‐6** and **POM‐7** were strongly toxic, but **POM‐1**, **POM‐2**, **POM‐5** and **POM‐11** were tolerated well up to 200 μM. Taking the cytotoxic effects, solubility issues and the activities reported in Tables 1 and 2 into account, complexes aurothiomalate (**Au‐2**), **Au‐12**, **Au‐33**, **Au‐34**, titanocene dichloride (**Ti‐1**), **Ag‐3** and **POM‐11** were selected for antiviral studies in SARS‐CoV‐2 infected Caco‐2 cells, with dosages adjusted accordingly.

**Figure 5:**
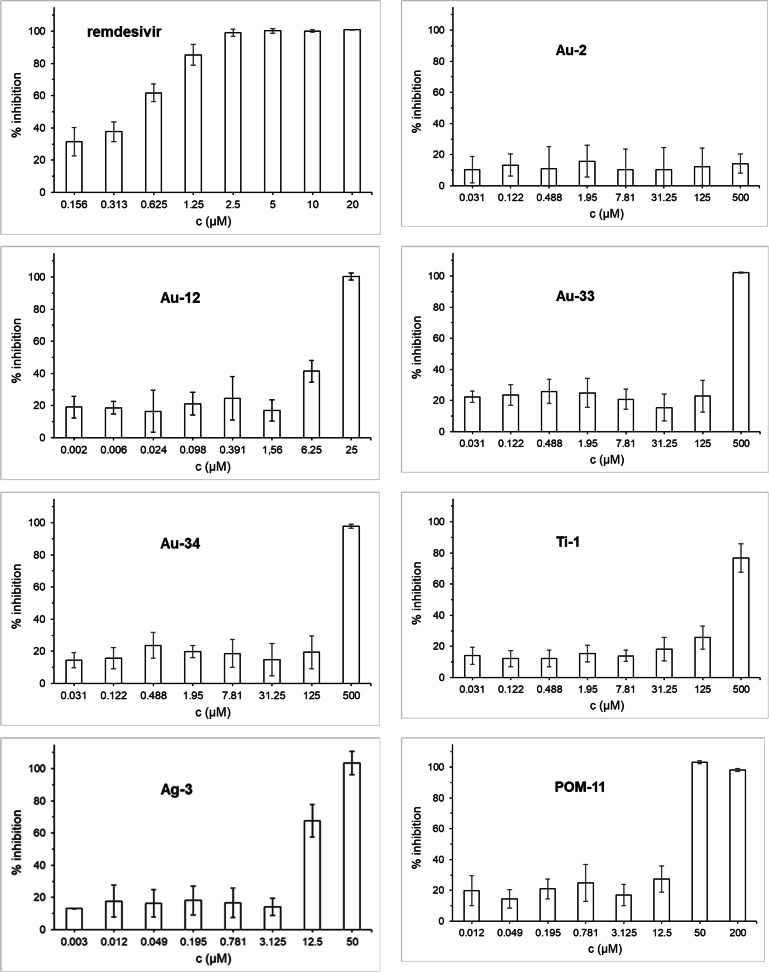
Antiviral effects of selected metallodrugs against SARS‐CoV‐2 replication in Caco‐2 cells after 24 h of exposure.

Confluent layers of Caco‐2 cells were infected with SARS‐CoV‐2 and the test compounds were added simultaneously with the virus. The antiviral activity against SARS‐CoV‐2 infection was determined after 24 h by immunohistochemistry of the viral S protein (Figure 5).

Remdesivir was used as a reference and efficiently inhibited viral replication at low micromolar concentrations. While the gold complex aurothiomalate (**Au‐2**) was not active, **Au‐12** at 6.25 and 25 μM concentrations efficiently inhibited the SARS‐CoV‐2. Gold compounds **Au‐33** and **Au‐34** were inactive at low dosages, but completely blocked SARS‐CoV‐2 at 500 μM. Titanocene dichloride (**Ti‐1**) was active at 125 and 500 μM, while **Ag‐3** and **POM‐11** showed strong effects already at low to mid‐micromolar concentrations (**Ag‐3**: 12.5 μM. **POM‐11**: 50 μM). Of note, **Ag‐3** caused cytotoxicity at 50 μM but was not toxic at 25 μM (see supporting info). Thus, the antiviral effects observed with this compound at 50 μM, but not those at 12.5 μM, are overlapping with host cell toxicity. Taken together, most of the selected metallodrugs showed clear antiviral effects against SARS‐CoV‐2, however, they did not reach the activity of the reference drug remdesivir.

## Discussion and Conclusions

More than 100 structurally diverse metal complexes were investigated against two relevant protein targets of coronaviruses, namely the S/ACE2 interaction and the protease PL^pro^.

Regarding the interference of the protein‐protein interaction of the RBD of the S protein with the ACE2 host receptor, within the mononuclear metal complexes only titanocene dichloride (**Ti‐1**) showed appreciable activity with an IC_50_ value in the low micromolar range. Results obtained with a few other examples, such as several complexes with nucleotide ligands, indicated that more active compounds might be identified after future structural optimisation. Curiously, the most pronounced activity against the S/ACE2 interaction was observed with polyoxometalates, some of which achieved IC_50_ values in the nanomolar concentration range.

These results are of particular interest as there are very few experimentally confirmed small molecule inhibitors of the entry of SARS‐CoV‐2 into host cells (e. g. arbidol, chloroquine) and their mechanism of action has not been fully elucidated yet.[^34^, ^36^, ^42^] The recently reported moderate activity of different gold compounds against the S/ACE2 interaction provided one of the first experimental confirmations of the inhibition of this protein‐protein interaction by small molecule drugs.^[6]^

The low micromolar activity of titanocene dichloride (**Ti‐1**) as inhibitor of the S/ACE2 interaction is of particular interest as this compound was inactive against the two types of PL^pro^, indicating that the interference with the protein‐protein interaction is the consequence of a specific mechanism. The sandwich structure appears to be of relevance for the effects, as the other investigated titanium species **Ti‐2**, titanium(IV) tetrachloride, was inactive in all used assays. Titanium(IV) complexes such as titanocene dichloride (**Ti‐1**) have been developed as anticancer agents, however, in a very few early reports also some antiviral effects were confirmed.^[43]^ In this study titanocene dichloride (**Ti‐1**) showed antiviral effects at concentrations higher than 100 μM. Whereas such activity appears too low for further development of titanocene dichloride (**Ti‐1**) as an antiviral drug, the results certainly suggest extended evaluation and careful optimization of titanium complexes as possible SARS‐CoV‐2 entry inhibitors. Previous preclinical and clinical studies on titanocene dichloride (**Ti‐1**) as an anticancer agent had revealed limitations in the stability of the drug, which should be considered in future drug design.^[44]^

Many of the studied gold metallodrugs exhibited strong activity against the protease PL^pro^ of both SARS‐CoV and SARS‐CoV‐2. Evaluation of structure‐activity‐relationships indicated a preference for complexes with good leaving groups (e. g. chloride) over compounds with firmly coordinated ligands such as dicarbene gold complexes of the type (NHC)_2_Au^+^, which were inactive. Exceptionally strong and selective activity against SARS‐CoV‐2 PL^pro^ was obtained with the gold(III)‐dithiocarbamato glycoconjugates **Au‐33** and **Au‐34**.

In addition to the strong inhibition of PL^pro^ and some low to moderate interference with the S/ACE2 protein‐protein interaction, several other mechanisms of gold metallodrugs may add to their potential as antiviral drugs. With the protease M^pro^, SARS‐CoV‐2 offers another moiety with a reactive cysteine that might be targeted by the soft Lewis‐acidic gold center of the complexes. In addition to that, anti‐inflammatory gold complexes such as auranofin are known to reduce cytokine expression and could therefore help to reduce the “cytokine storm” that SARS‐CoV‐2 causes in the infected cells. In fact, for auranofin the inhibition of SARS‐CoV‐2 replication in cells and the reduction of the expression of SARS‐COV‐2‐induced cytokines has been recently confirmed.^[16]^ In a recent screening of more than 1000 FDA‐approved drugs, auranofin was among the 44 most promising hit compounds, however, in the second round of the screening it was among the 32 compounds that were excluded because of cytotoxicity.^[45]^ Keeping this in mind, the cytotoxic effects against almost confluent cell layers as used in antiviral assays were evaluated for the most active PL^pro^ inhibiting gold complexes after 24 h. In fact most of the gold complexes showed too strong toxicity (see supporting information), however, for four compounds very low or missing toxicity against the Caco‐2 cell line was noted and these four complexes were selected for the SARS‐CoV‐2 antiviral assays. Among those, the gold(I) NHC complex **Au‐12** showed very promising activity in the low micromolar concentration range and the two gold(III)‐dithiocarbamato complexes **Au‐33** and **Au‐34** were strongly active at the highest applied non toxic dosage. Taken together, further evaluation of gold compounds as SARS‐CoV‐2 antivirals is suggested in combination with a critical evaluation of their toxicity against host cells.^[15]^ Besides toxicity against host cells, the insufficient “transfer” of the very effective PL^pro^ inhibition into strong antiviral activity in the cell culture model might provide a limitation for gold complexes as SARS‐CoV‐2 antiviral agents that requires attention and might be overcome with appropriate targeting strategies.

All of the three studied silver complexes triggered strong inhibition of SARS‐CoV‐2 PL^pro^ but were much less active against the enzyme from SARS‐CoV. The activity appears to be strongly connected to the silver(I) ion, as silver nitrate had a comparable activity to the other two compounds. The studied silver complexes include silver sulfadiazine (**Ag‐2**), which combines a silver ion with the antibacterial drug sulfadiazine and is used as a slow silver‐releasing topical antimicrobial therapeutic agent.^[46]^ Antiviral properties of silver sulfadiazine and a silver complex containing a NHC ligand have already been reported against the herpes virus as well as HIV, respectively.[^7^, ^47^] The antibacterial properties of silver have been well known for a long time and are used in many areas.^[48]^ Antiviral effects have been less frequently explored, however, it is interesting to note that, for silver nanoparticles antiviral activity against SARS‐CoV‐2 was confirmed.^[49]^ After toxicity evaluation, the silver NHC complex **Ag‐3** was studied for antiviral effects against SARS‐CoV‐2 and recorded strong activity at 12.5 μM and 50 μM. Of note, **Ag‐3** was not toxic up to 25 μM, however, at 50 μM cytotoxic effects were observed and therefore the results obtained at the highest concentration need to be interpreted with caution. Same as for the gold complexes, cytotoxicity against host cells is a critical and without structural optimisation maybe even limiting factor.

The POMs investigated in this report can be roughly grouped into three different classes:

- classical POMs (**POM‐3** to **POM‐5**) characterized by high charge density and highly hydrophilic surface;
- hybrid POMs (**POM‐6** to **POM‐10**), which are classical POMs functionalized by organic ligands
- polyoxopalladates (POPs), which are polynuclear palladium‐oxo clusters with (**POM‐11**) or without (**POM‐1**, **POM‐2**) organic ligand functionalization.

The classical POMs generally showed lower activities against PL^pro^ compared to the other POMs, and this might be a consequence of the high charge densities and hydrophilicities. However, with the exception of **POM‐5**, they were similarly effective as the other POMs with regards to the inhibition of the S/ACE2 interaction. Notably, for **POM‐3** to **POM‐5**, antiviral activity against the Zika virus by entry blockage has been confirmed before.^[25]^ The hydrophilic and charged surfaces could be of benefit in interfering with the protein‐protein interaction between S and ACE2. Among the hybrid POMs, **POM‐6** and **POM‐7** exhibited strong activities both against the S/ACE2 interaction as well as the protease PL^pro^, whereas **POM‐8** to **POM‐10** were much less active in both assays. This could be attributed to the nature of the external organic functional groups attached. As demonstrated in previous studies, in going from a hydrophilic exterior to a hydrophobic one, the biological activity of POMs can be switched on and, by tuning the hydrophobic exterior of the POM clusters, their antibacterial activity could be modulated.^[50]^ **POM‐6** and **POM‐7**, in particular, have shown good antibacterial and antitumoral activities.^[31]^ However, **POM‐8** to **POM‐10** have phenyl groups with carboxylate, azido and bromo functionalities in the para position, which makes the exterior less hydrophobic and more hydrophilic and therefore could explain the substantially lower activities. As for the polyoxopalladates (POPs), both the organically functionalized (**POM‐11**) and non‐functionalized (**POM‐1** and **POM‐2**) POPs displayed good results in both assays. This is the first report on biomedical properties of the recently discovered **POM‐11**. On the other hand, **POM‐1** and **POM‐2** were already shown to be effective as anti‐cancer agents.^[27]^ These results demonstrate that POMs are highly attractive for biomedical applications, as long as they are solution‐stable at physiological pH (ideally proven by NMR). Several studies in the literature are based on POMs that do not fulfill such conditions, and hence it is likely that POM decomposition/transformation products contribute to the observed bioactivity.^[23]^

**Figure 6:**
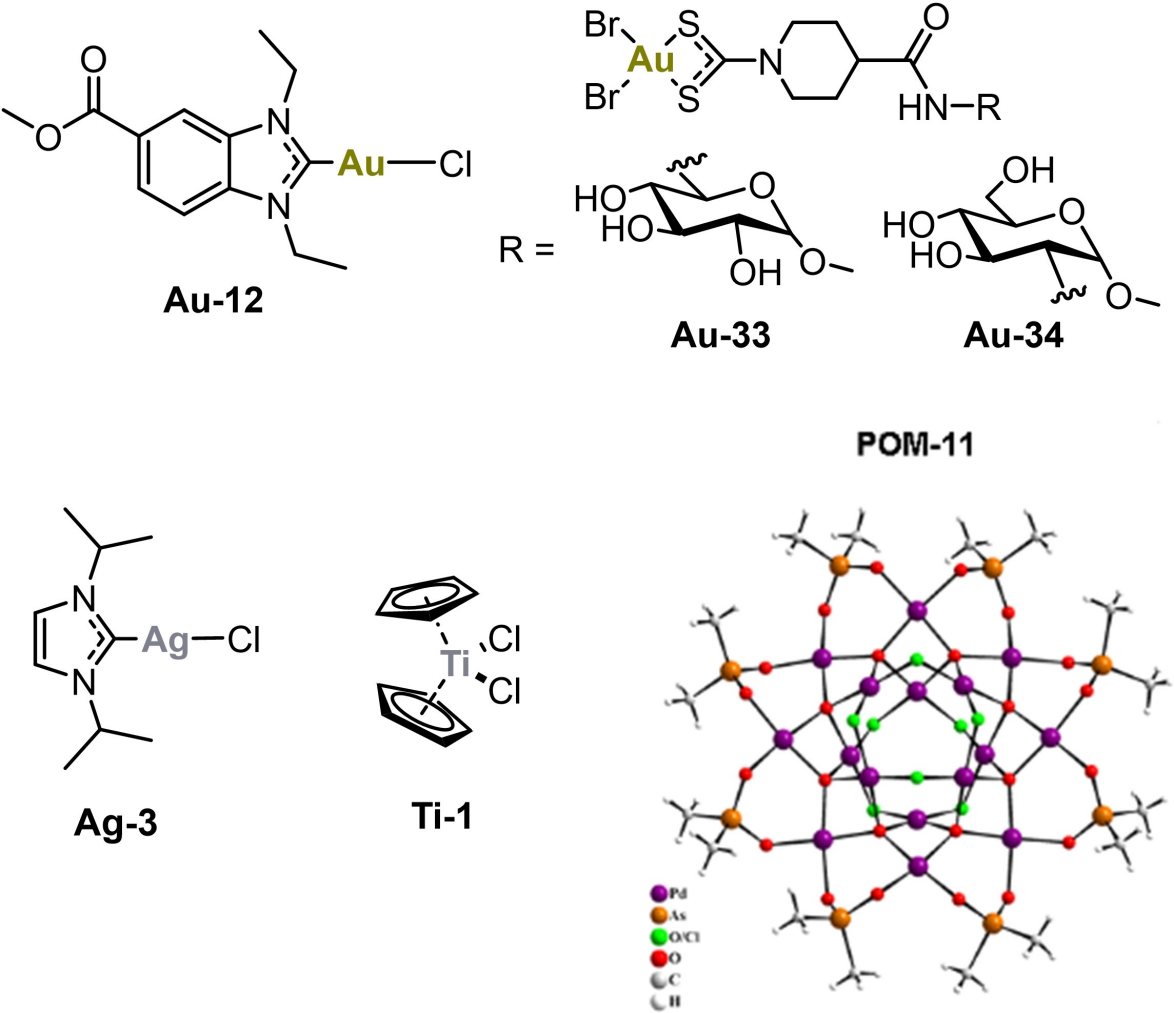
Most promising examples with antiviral activity against SARS‐CoV‐2.

In summary, the metallodrug profiling of more than 100 individual complexes afforded strong inhibitors of the S/ACE2 interaction and in particular of the PL^pro^ enzymatic activity. After toxicity evaluation, several complexes were selected for antiviral assays in SARS‐CoV‐2 infected Caco‐2 cells. The most promising results were obtained with **Au‐12**, **Ag‐3** and **POM‐11**, which showed antiviral activity starting at low micromolar concentrations (Figure 6). In addition, **Au‐33**, **Au‐34** and titanocene dichloride (**Ti‐1**) strongly blocked viral replication at a high dosage of 500 μM.

Taken together, the results of this study indicate that antiviral metallodrugs against SARS‐CoV‐2 can be designed in future studies. In particular, complexes based on silver or gold, titanocenes, or POMs might be developed to new lead compounds. From a translational perspective, in particular the metal NHC complexes **Au‐12** and **Ag‐3** are interesting as they represent compounds, in which a neutral organic ligand is relatively stably coordinated to a metal center, while the remaining chlorido ligands are required to facilitate the inhibition of the target cysteine protease PL^pro^. Further in vitro and in vivo studies are suggested in order to characterize the potential as SARS‐CoV‐2 antiviral agents in more detail. Regarding the gold complex **Au‐12**, this should include the evaluation of possible effects on host cell cytokine release.^[16]^

## Experimental

### Compounds

Some tested compounds are commercially available and they were purchased from Sigma Aldrich (sodium aurothiomalate hydrate, gold(III) chloride hydrate, chloro(triethylphosphine)gold(I), chloro(triphenylphosphine)gold(I), thiomersal), Acros Organics (silver sulfadiazine, titanocene dichoride, cisplatin, disulfiram), Adipogen‐Biomol (chloroquine diphosphate) and Enzo Life Sciences (auranofin). The following complexes were prepared as previously described in the literature: **Au‐7** ^[51]^, **Au‐8** ^[52]^, **Au‐9**, **Au‐11** ^[53]^, **Au10** ^[54]^, **Au‐12**, **Au‐13** ^[55]^, **Au‐15** ^[52]^, **Au‐20**, **Au‐25**, **Au‐26** ^[56]^, **Au‐27** ^[57]^, **Au‐31**–**Au‐32** ^[55]^, **Au‐33**, **Au‐34** ^[58]^, **Ru‐1**, **Ru‐4**–**Ru‐8**,^[59]^, **Ru‐10** ^[60]^, **Ru‐11** ^[61]^, **Ru‐12** ^[62]^, **Ru‐13^[63]^**, **Ru‐14**, **Ru‐15**, **Ru‐16** ^[64]^, **Mn‐1**, **Mn‐2** ^[65]^, **Re‐1** ^[66]^, **Re‐2** ^[67]^, **Pt‐2** ^[68]^, **Pt‐3**, **Pt‐4**, **Pd‐1**, **Pd‐2** ^[69]^, **Fe‐1**, **Fe‐2**, **Fe‐3**, **Fe‐4** ^[70]^, **Fe‐5** ^[71]^, **Fe‐6** ^[72]^, **Fe‐7**, **Fe‐8** ^[73]^ **Fe‐9** ^[74]^, **Fe‐10** ^[75]^, **Fe‐11** ^[76]^, **Fe‐13** ^[77]^, **POM‐1**, **POM‐2** ^[27]^, **POM‐3** ^[28]^, **POM‐4** ^[29]^, **POM‐5** ^[30]^, **POM‐6**, **POM7** ^[31]^, **POM‐8**, **POM‐9**, **POM‐10** ^[32]^, **POM‐11** ^[33]^. For preparation and characterization of all other complexes see the supporting information.

### S/ACE2 Interaction Assay

The inhibition of the S‐ACE2 interaction was measured using the SARS‐CoV‐2 Inhibitor Screening Assay kit (Adipogen, Cat. N° AG‐48B‐0001‐KI01). All reagents were used from the same kit during individual experiments and the experiments were performed using the manufacturer's protocol. Briefly, the SARS‐CoV‐2 Spike S receptor binding domain (RBD): Fc (human) (rec.) (SPIKE) was reconstituted to 0.1 mg/mL with deionized water. This was further diluted to a working concentration of 1 μg/mL in phosphate buffered saline (PBS) and used freshly. The assay plate was coated with 100 μL/well of SPIKE, covered with a plastic film and kept at 4 °C overnight. The liquid was aspirated and any remaining liquid was removed by blotting against clean absorbent papers. The plate was blocked using 200 μL of Blocking Buffer per well for 2 h at room temperature. The liquid was aspirated and the wells were washed with 1X Washing Buffer (300 μL×3 times). All liquid was aspirated and excess liquid was removed by blotting against clean absorbent papers. The inhibitors (metal complexes, controls, reference) were diluted in Inhibitor Mix Solution (IMS), which was prepared using ACE2 (human) (rec.) (Biotin) (ACE2) (stock solution 0.1 mg/mL) to the working concentration of 0.5 μg/mL in 1X ELISA Buffer. Fresh stock solutions of the inhibitors were made in DMSO or water (**Au‐2**, **Ag‐1**, **Ag‐2** and POMs) and the final concentration of the vehicle in the wells was 0.5 %. The IMS‐diluted inhibitors were added to the wells (100 μL/well). The final concentrations of the inhibitors were 20 μM for the single dosage screening and in the range of 0.1 to 200 μM for determination of IC_50_ values. The negative control wells were also treated with 0.5 % DMSO in IMS. The plate was covered with a plastic film and incubated at 37 °C for 1 h after which the aspiration/wash step was repeated. Next, horseradish peroxidase‐labeled streptavidin (HRP) was reconstituted with 100 μL of 1X ELISA Buffer and further diluted to a working concentration by adding 50 μL in 10 mL of 1X ELISA Buffer (1 : 200 dilution). It was covered with a plastic film and incubated at RT for 1 h. Following this, the aspiration/wash step as described earlier was repeated. Substrate development was conducted by the addition of 100 μL of ready‐to‐use 3,3′,5,5′‐tetramethylbenzidine (TMB) to each well for 5 minutes at RT. The reaction was stopped by adding 50 μL of a stop solution (H_2_SO_4_ 5 M). The OD was measured at 450 nm using a Perkin Elmer Victor X4 microplate reader. The individual absorbance value of the blank well was subtracted from the other absorbance values and the percentage of the remaining activity was calculated with respect to the untreated control values. Data fitting was done using Origin 2018 using sigmoidal fitting with Hill1 fitting curve. All treatments were done in duplicates and two independent experiments were performed.

### SARS‐CoV and SARS‐CoV‐2 PL^pro^ Inhibition

The inhibitor candidate compounds were prepared as fresh stock solutions in DMSO or water and diluted hundredfold with HEPES buffer (50 mM HEPES, pH 7.5, 0.1 mg/mL bovine serum albumin, 0.1 v. % Triton‐X100) to micromolar concentrations. Volumes of 50 μL of 200 nM His_6_‐SARS‐CoV‐1 PLpro (SouthBayBio) or of 200 nM SARS‐CoV‐2 PLpro (Elabscience) in HEPES buffer or blank HEPES buffer (negative control) were added to the wells of a black 96‐well microtiter plate (Nunclon, Nunc). Volumes of 50 μL of the inhibitor solutions or 1 % DMSO/water (**Au‐2**, **Ag‐1**, **Ag‐2** and POMs) in HEPES buffer (positive control) were added. The resulting solutions (100 nM SARS‐CoV PL^pro^ or 100 nM SARS‐CoV‐2 PL^pro^ 0.5 % DMSO, 10 μM/1 μM single dose or IC_50_ 0.001–50 μM test compound or blank HEPES buffer) were mixed and incubated at 37 °C for one hour. A volume of 100 μL 100 μM Z‐Arg‐Leu‐Arg‐Gly‐Gly‐AMC (Bachem Bioscience) was added to all wells. The resulting solutions were mixed and the fluorescence emission was measured immediately every 30 s for 10 min (λ_exc_=355 nm; λ_em_=460 nm) at 37 °C using a Victor^TM^ X4 Perkin Elmer 2030 multilabel reader. The increase of emission over time followed a linear trend (r^2^>0.97) and the enzymatic activities were calculated as the slope thereof. The IC_50_ values were calculated as the concentration of the inhibitor that was required to decrease the enzymatic activity to 50 % of the positive control. The wells containing the negative control were used to confirm the absence of false positive results by reaction of the inhibitor compound with the fluorogenic substrate. All experiments were done in duplicates and two independent experiments were performed.

### Antiviral effects against SARS‐CoV‐2

#### Cell lines

Caco‐2 cells were grown at 37 °C in Minimal Essential Medium (MEM) supplemented with 10 % fetal bovine serum (FBS) and containing 100 IU/mL penicillin and 100 μg/mL streptomycin. All culture reagents were purchased from Sigma. The cells have been tested negative for mycoplasma infection.

#### Virus isolation and preparation

SARS‐CoV‐2/FFM7 was isolated from naso‐pharyngeal swabs of COVID‐19 patients using human colon carcinoma cell line CaCo‐2. SARS‐CoV‐2 stocks used in the experiments had undergone two passages on CaCo‐2 cells and were stored at −80 °C. Virus titers were determined as TCID50/mL in confluent cells in 96‐well microtiter plates. The isolate was authenticated and the sequence is publicly available at GISAID.org.

### Antiviral activity of metal‐based compounds

Confluent layers of CaCo‐2 were infected with SARS‐CoV‐2/FFM7 at a MOI of 0.01. The tested compounds were added simultaneously with virus and incubated in MEM supplemented with 1 % FBS. Immunohistochemistry of viral S protein was performed after 24 h post infection to determine antiviral activity against SARS‐CoV‐2 infection.

### Immunohistochemistry

Cells were fixed with acetone : methanol (40 : 60) solution and immunostaining was performed using a monoclonal antibody directed against the S protein of SARS‐CoV‐2 (1 : 1500, Sinobiological), which was detected with a peroxidase‐conjugated anti‐rabbit secondary antibody (1 : 1,000, Dianova), followed by addition of AEC substrate. The S positive area was scanned and quantified by automated plate reader. The results were expressed as percentage of infection relative to virus control which received no drug.

## General contribution list

Maria Gil‐Moles, Sebastian Türck and Uttara Basu: chemical synthesis, enzymatic assays, data analysis, manuscript preparation; Andrea Pettenuzzo, Saurav Bhattacharya, Ananthu Rajan, Xiang Ma, Rolf Büssing, Jessica Wölker, Hilke Burmeister, Henrik Hoffmeister, Pia Schneeberg, Andre Prause, Josephine Kusi‐Nimarko, Storm Hassell‐Hart, Andrew McGown, Daniel Guest, Yan Lin, Anna Notaro, Robin Vinck, Johannes Karges, Kun Peng, Xue Qing, Xing Wang, Joanna Skiba, Łukasz Szczupak: chemical synthesis, Petra Lippmann: toxicity assays; Kevin Cariou, Konrad Kowalski, Ulrich Schatzschneider, Catherine Hemmert, Heinz Gornitzka, Elena R. Milaeva, Alexey A. Nazarov, Gilles Gasser, John Spencer, Luca Ronconi, Ulrich Kortz: supervision, manuscript preparation; Jindrich Cinatl, Denisa Bojkova: antiviral assays, data analysis; Ingo Ott: project leader, supervision, data analysis, manuscript preparation.

## Conflict of interest

The authors declare no conflict of interest.

## Supporting information

As a service to our authors and readers, this journal provides supporting information supplied by the authors. Such materials are peer reviewed and may be re‐organized for online delivery, but are not copy‐edited or typeset. Technical support issues arising from supporting information (other than missing files) should be addressed to the authors.

Supporting Information
