# Supplementary material for: Metallodrug Profiling against SARS‐CoV‐2 Target Proteins Identifies Highly Potent Inhibitors of the S/ACE2 interaction and the Papain‐like Protease PLpro
Source: Chemistry. 2021 Nov 23;27(71):17928–40. doi: 10.1002/chem.202103258 (PMC8653295; doi:10.1002/chem.202103258)
Supplement: Supplementary file 1 — Supporting Information [file CHEM-27-17928-s001.pdf]

Chemistry–A European Journal

Supporting Information

Metallodrug Profiling against SARS-CoV-2 Target Proteins Identifies Highly Potent Inhibitors of the S/ACE2 interaction and the Papain-like Protease PL^{pro}

Maria Gil-Moles, Sebastian Türck, Uttara Basu, Andrea Pettenuzzo, Saurav Bhattacharya, Ananthu Rajan, Xiang Ma, Rolf Büssing, Jessica Wölker, Hilke Burmeister, Henrik Hoffmeister, Pia Schneeberg, Andre Prause, Petra Lippmann, Josephine Kusi-Nimarko, Storm Hassell-Hart, Andrew McGown, Daniel Guest, Yan Lin, Anna Notaro, Robin Vinck, Johannes Karges, Kevin Cariou, Kun Peng, Xue Qin, Xing Wang, Joanna Skiba, Łukasz Szczupak, Konrad Kowalski, Ulrich Schatzschneider, Catherine Hemmert, Heinz Gornitzka, Elena R. Milaeva, Alexey A. Nazarov, Gilles Gasser, John Spencer, Luca Ronconi, Ulrich Kortz, Jindrich Cinatl, Denisa Bojkova, and Ingo Ott*

Supporting Information

Table of contents

1) Synthesis of gold compounds
2) Synthesis of ruthenium compounds
3) Synthesis of rhodium compounds
4) Synthesis of iron compounds
5) Synthesis of silver complex Ag-3
6) Tables S1 to S3 (Results of the inhibitor assays)
7) Toxicity against cells

1) Synthesis of gold compounds

General for compounds Au-6 - Au-29

Chemicals and reagents were obtained from Sigma-Aldrich, TCI, Alfa Aesar and ACROS unless otherwise noted. NMR spectra were recorded on a Bruker DRX-400 AS, a AV III HD 500 NMR or a AVII 600 spectrometer; Electrospray ionization (ESI) mass spectra were recorded on a Finnigan MAT95 XL, a LTQ-OrbitrapVelos linear ion trap coupled to an Orbitrap mass analyser (ThermoFisher Scientific) or an expression^{L} CMS spectrometer (Advion). Elemental analyses were conducted on a Flash EA1112 apparatus.

(1,3-Dimethylbenzimidazol-2-ylidene)((4-methoxyphenyl)ethynyl)gold(I) **Au-6**:

1-Ethynyl-4-methoxybenzene (87.3 mg, 0.661 mmol, 1 equiv.) and potassium hydroxide (222.3 mg, 3.962 mmol,6 equiv.) were dissolved in methanol (20 mL) and the mixture was stirred for 10 min at 50 °C. Afterwards, chlorido(1,3-dimethyl-benzimidazol-2-ylidene)gold(I) (250.0 mg, 0.660 mmol, 1 equiv.) was dissolved in dichloromethane (1 mL) and added to the mixture. The mixture was stirred for 4 h at 65 °C and then for 60 h at room temperature under protection from light. Afterwards, the solvent was removed under vacuum, the residue dissolved in dichloromethane, and filtered into a separating funnel. The solution was washed with a potassium hydroxide solution (20 g/L). The complex was dried under vacuum at 40 °C. Yield: 58% yellow powder. ^{1}H NMR (400 MHz, DMSO-d_6): δ (ppm) = 7.73-7.79 (m, 2H, ArH), 7.46-7.51 (m, 2H, ArH), 7.20-7.25 (m, 2H, ArH), 6.81-6.84 (m, 2H, ArH), 4.03 (s, 6H, CH_3), 3.74 (s, 3H, OCH_3); ^{13}C NMR (101 MHz, DMSO-d_6): **δ** (ppm) = 193.19 (ArC_{quart}), 157.59 ($ArC_{quart.}$), 133.49 (2 $ArC_{quart.}$), 132.38 (2 ArC), 131.00 ($ArC_{quart.}$), 123.98 (2 ArC), 118.30 ($ArC_{quart.}$), 113.80 (2A ArC), 111.03 (2 ArC), 103.80 ($ArC_{quart.}$), 50.01 (OCH_3), 34.51 (CH_3); MS (ESI$^+$): *m*/*z* = 475.1 $[M+H]^+$, 817.7 $[M+NHC-Au]^+$; elemental analysis for $C_{18}H_{17}AuN_2O$ (%calc. / %found): C (45.58 / 45.26), H (3.36 / 3.39), N (5.91 / 5.81).

Chloro(1,3-dimethyl-4-phenylimidazol-2-ylidene)gold(I) **Au-14**

1,3-Dimethyl-4-phenyl-1*H*-imidazol-2-ium iodide (150.1 mg, 0.5 mmol) was treated with 0.5 equivalents of silver(I) oxide (57.8 mg, 0.25 mmol) in a mixture of dichloromethane and ethanol (1:1) overnight. Afterwards, chloro(dimethylsulfide)gold(I) (147.2 mg, 0.5 mmol) was added to the suspension, which was left under vigorous stirring overnight. The suspension was filtered through Celite and then evaporated under reduced pressure. The white residue was purified by column chromatography on silica with dichloromethane as the eluent to obtain a pure product. Yield: 81% white powder

^{1}H NMR (600 MHz, $CDCl_3$): δ (ppm) = 7.52-7.46 (m, 3H; $ArH_{meta+para}$), 7.39-7.31 (m, 2H; ArH_{ortho}), 6.91 (s, 1H; ImH5), 3.87 (s, 3H; CH_3), 3.78 (s, 3H; CH_3); ^{13}C NMR (151 MHz, $CDCl_3$): δ (ppm) = 172.36 (Im-C2), 135.23 (Im-C4quat.), 129.70 (Ph-CH_{para}), 129.16 (Ph-CH_{ortho}), 129.10 (Ph-CH_{meta}), 127.31 (Im-$C4_{quat.}$), 119.32 (Im-C5), 38.33 (CH_3), 36.63 (CH_3); MS (ESI^+): m/z = 401.2 $[M\text{-}Cl+CH_3OH]^+$, 773.4 $[2M\text{-}Cl]^+$; elemental analysis for $C_{11}H_{12}AuN_2Cl$ (%calc. / %found): C (32.65 / 32.82), H (2.99 / 2.77), N (6.92 / 6.62).

Chloro(1,3-diisopropyl-4-phenylimidazol-2-ylidene)gold(I) **Au-16**

1,3-Diisopropyl-4-phenyl-1*H*-imidazol-2-ium iodide (207.8 mg, 0.58 mmol) was treated with 0.5 equivalents of silver(I) oxide (65.4 mg, 0.28 mmol) in a mixture of dichloromethane and ethanol (1:1) overnight. Then, chloro(dimethylsulfide)gold(I) (172.0 mg, 0,56 mmol) was added to the suspension and again left under vigorous stirring overnight. The obtained suspension was filtered over celite and afterwards evaporated under reduced pressure. The pale orange residue was purified by column chromatography on silica with dichloromethane to obtain the pure product. Yield: 70% pale orange powder. ^{1}H NMR (500 MHz, $CDCl_3$): δ (ppm) = 7.53-7.44 (m, 3H; $ArH_{meta+para}$), 7.36-7.28 (m, 2H; ArH_{ortho}), 6.89 (s, 1H; ImH5), 5.18 (hept, J = 6.8 Hz, 1H; CH), 4.53 (hept, J = 6.9 Hz, 1H; CH), 1.72 (d, J = 6.9 Hz, 6H; CH_3), 1.51 (d, J = 6.7 Hz, 6H; CH_3); ^{13}C NMR (126 MHz, $CDCl_3$) δ (ppm) = 167.27 (Im-C2), 134.92 (Im-$C4_{quat.}$), 130.01 (Ph-CH_{ortho}), 129.67 (Ph-CH_{para}), 128.93 (Ph-CH_{meta}), 128.07 (Im-$C4_{quat.}$), 113.86 (Im-C5), 54.88 (CH), 50.60 (CH), 24.23 (CH_3), 23.36 (CH_3); MS (ESI^+): m / z = 457.3 $[M\text{-}Cl+CH_3OH]^+$, 885.5 $[M\text{-}Cl+M]^+$; MS (ESI^-): m / z = 267.0 $[AuCl_2]^-$; elemental analysis for $C_{15}H_{20}AuN_2Cl$ (%calc. / %found): C (36.09 / 36.31), H (3.73 / 3.64), N (6.47 / 6.30).

Chloro(1,3-dibenzyl-4-phenylimidazol-2-ylidene)gold(I) **Au17**:

1,3-Dibenzyl-4-phenyl-1*H*-imidazol-2-iumbromide (149.3 mg, 0.49 mmol) was treated with 0.5 equivalents of silver(I) oxide (58.3 mg, 0.25 mmol) in a mixture of dichloromethane and ethanol (1:1) overnight. Afterwards chloro(dimethylsulfide)gold(I) (144.8 mg, 0.49 mmol) was added to the suspension and again left under vigorous stirring overnight. The obtained suspension was

filtered over celite and afterwards evaporated under reduced pressure until it was completely dry. The white residue was purified by column chromatography on silica with dichloromethane to obtain the pure product. Yield: 20% white powder. ^{1}H NMR (600 MHz, $CDCl_3$): δ (ppm) = 7.42-7.36 (m, 6H; ArH), 7.35-7.30 (m, 2H; ArH), 7.25-7.19 (m, 3H; ArH), 7.12-7.08 (m, 2H; ArH), 7.02-6.98 (m, 2H; ArH), 6.84 (s, 1H; ImH5), 5.45 (s, 2H; CH_2), 5.42 (s, 2H; CH_2); ^{13}C NMR (151 MHz, $CDCl_3$): δ (ppm) = 172.29 (Im-C2H), 135.67 (Ph-$C1_{quat.}$), 135.49 (Benz-$C1_{quat.}$), 134.88 (Benz-$C1_{quat.}$), 129.75 (Ph-CH_{para}), 129.59 (Ph-CH_{ortho}), 129.19 (Ph-CH_{meta}), 128.87 (Benz-CH_{para}), 128.80 (Benz-CH_{meta}), 128.69 (Benz-CH_{meta}), 128.26 (Benz-CH_{ortho}), 128.11 (Benz-CH_{para}), 127.25 (Im-$C4_{quat.}$), 127.19 (Benz-CH_{ortho}), 118.60 (Im-C5), 55.52 (CH_2), 52.59 (CH_2); MS (ESI$^+$): m / z = 553.2 $[M\text{-}Cl+CH_3OH]^+$, 753.4 $[M+Au]^+$; MS (ESI$^-$): m / z = 555.0 $[M\text{-}H]^-$, 266.8 $[AuCl_2]^-$; elemental analysis for $C_{23}H_{20}AuN_2Cl$ (%calc. / %found): C (49.61 / 49.59.), H (3.62 / 3.45), N (5.03 / 4.89).

1-(2-((2,4-dichlorobenzyl)oxy)-2-(2,4-dichlorophenyl)ethyl)-3-ethylimidazol-2-ylidene)gold(I) chloride **Au-18**

1-(2-((2,4-dichlorobenzyl)oxy)-2-(2,4-dichlorophenyl)ethyl)-3-ethyl-imidazolium iodide (0.4 mmol) was treated with 0.5 equivalents of silver oxide (0.0463 g, 0.2 mmol) for 4 h in dichloromethane under vigorous stirring at room temperature in the dark. After this period dimethylsulfidegold(I) (0.141 g, 0.48 mmol) was added and the reaction was stirred overnight. The obtained suspension was filtered over celite and the solvent was evaporated under reduced pressure to give a foam. The resulting foam was washed with ether under vigorous stirring and filtered to obtain the pure compound. Yield: 78% white solid. ^{1}H NMR (400 MHz, DMSO-d_6): δ (ppm) = 7.65 (d, 1H, J = 2.07 Hz, ImH), 7.56-7.35 (m, 7H, ImH + $PhCl_2$), 5.23 (dd, 1H, J = 7.77 Hz, J = 3.70 Hz, CH), 4.49 - 4.25 (m, 4H, CH_2), 4.09 (q, 2H, J = 7.32 Hz, CH_2), 1.33 (t, 3H, J = 7.32 Hz, CH_3); ^{13}C NMR (100 MHz, DMSO-d_6): δ (ppm) = 168.13 (ImC-2),134.17-133.14 (ArC), 131.01-120.37 (ArC + ImC4 + ImC5), 76.67 (CH), 67.27 (CH_2), 54.22 (CH_2), 46.28 (CH_2), 16.35 (CH_3); MS (ESI+): m/z = 443.2 $[M-AuCl]^+$, 641.3 $[M-Cl]^+$, 673.2 $[M-Cl+MeOH]^+$; elemental analysis for $C_{20}H_{18}Au_1Cl_5N_2O_2$ (%calc. / %found): C (35.50 / 35.47), H (2.68 / 2.62), N (4.14 / 3.91).

(1-(((2R,4S)-4-((4-(4-acetylpiperazin-1-yl)phenoxy)methyl)-2-(2,4-dichlorophenyl)-1,3-dioxolan-2-yl)methyl)-3-ethyl-1H-imidazol-3-ium-2-yl)gold(I) chloride **Au-19**

1-(((2R,4S)-4-((4-(4-acetylpiperazin-1-yl)phenoxy)methyl)-2-(2,4-dichlorophenyl)-1,3-dioxolan-2-yl)methyl)-3-ethyl-1H-imidazol-3-ium iodide (0.4 mmol) was treated with 0.5 equivalents of Ag_2O (0.0463 g, 0.2 mmol) overnight in dichloromethane under vigorous stirring at room temperature in the dark. After this period dimethylsulfidegold(I) (0.141 g, 0.48 mmol) was added and the reaction was stirred 2 days. The obtained suspension was filtered over

celite and the solvent was evaporated under reduced pressure to give a foam. The resulting foam was washed with ether under vigorous stirring and filtered to obtain the pure compound. Yield: 61% white solid. ^{1}H NMR (400 MHz, DMSO-d_6): δ (ppm) = 7.62-7.30 (m, 5H, ImH + $PhCl_2$), 6.92-6.83 (AA'BB', 4H, HAr), 4.70 (m, 2H, CH_2), 4.38 (m, 1H, CH), 4.03-3.543.36 (m, 10H, piperazineCH_2+dioxalanCH_2+ CH_2), 2.98 (m, 4H, piperazineCH_2), 2.78 (s, 3H, CH_3), 1.26 (t, 3H, J = 7.04 Hz, CH_3); ^{13}C NMR (100 MHz, DMSO-d_6): δ (ppm) = 169.9 (ImC2) 168.04 (C=O), 151.9-145.5 (CAr-N + CAr-O), 134.6-132.7 (ArC), 130.8-115.0 (CH-Ar + ImC4 +ImC5), 107.2 (C-dioxalan), 74.3 (CH-dioxalan) 67.9-40.6 (CH_2-piperazine+CH_2-dioxalan+CH_2), 20.8 (CH_3-C=O), 15.9 (CH_3); MS (ESI^+): m/z = 559.3 $[M-AuCl]^+$, 755.3 [M-Cl]; elemental analysis calculated for $C_{28}H_{32}AuCl_3N_4O_4$: (%calc / %found): C (42.47 / 42.44), H (4.07 / 4.02), N (7.07 / 6.79).

Di-(1,3-dimethylbenzimidazol-2-ylidene)gold(I)iodide **Au-21**

Chloro(1,3-dimethylbenzimidazol-2-ylidene)gold(I) (45.2 mg, 0.12 mmol) was treated with 1 equivalent of 1,3-dimethylbenzimidazoliumiodide (33.0 mg, 0.12 mmol) and 1.5 equivalents of K_2CO_3 in a mixture of dichloromethane and methanol (1:1) for two days under vigorous stirring at room temperature. The obtained suspension was filtered over celite and the clear solution was evaporated under reduced pressure. The white resultant was dissolved in dichloromethane (2 mL) and then transferred into diethyl ether (30 mL) in an ultrasonic bath. The suspension was filtered to obtain the pure product. Yield: 95% white powder. ^{1}H NMR (600 MHz, DMSO-d_6): δ (ppm) = 7.90-7.84 (m, 4H; CH4,7), 7.60-7.54 (m, 4H; CH5,6), 4.19 (s, 12H; CH_3); ^{13}C NMR (151 MHz, DMSO-d_6): δ (ppm) = 190.13 (C2), 133.50 (ArC), 124.55 (ArC), 112.05 (ArC), 34.74 (CH_3); MS (ESI^+): m / z = 489.2 $[M–I]^+$; MS (ESI^-): m / z = 126.9 $[I]^-$; elemental analysis for $C_{18}H_{20}AuN_4I$ (%calc. / %found): C (35.08 / 34.90), H (3.27 / 3.29), N (8.09 / 8.71).

Di-(1,3-diisopropylbenzimidazol-2-ylidene)gold(I)bromide **Au-22**

Chloro(1,3-diisopropylbenzimidazol-2-ylidene)gold(I) (94.4 mg, 0.22 mmol) was treated with 1 equivalent of 1,3-diisopropylbenzimidazoliumbromide (79.1 mg, 0.28 mmol) and 1.5 equivalents of K_2CO_3 in a mixture of dichloromethane and methanol (1:1) for two days under vigorous stirring at room temperature. The obtained suspension was filtered over celite and the clear solution was evaporated under reduced pressure. The white resultant was dissolved in dichloromethane (2 mL) and then transferred into diethyl ether (30 mL) in an ultrasonic bath. The suspension was filtered to obtain the pure product. Yield: 78% white powder. ^{1}H NMR (500 MHz, $CDCl_3$): δ (ppm) = 7.81-7.73 (m, 4H; CH4,7), 7.52-7.44 (m, 4H; CH5,6), 5.44 (hept, J = 7.0 Hz, 4H; CH), 1.88 (d, J = 7.0 Hz, 24H; CH_3); ^{13}C NMR (126 MHz, $CDCl_3$): δ (ppm) = 186.86 (C2), 132.62 (ArC), 124.79 (ArC), 113.20 (ArC), 53.82 (CH), 22.61 (CH_3); MS (ESI^+):

m / z = 601,4 [M-Br]$^+$; elemental analysis for $C_{26}H_{36}AuN_4Br$ (%calc. / %found): C (45.83 / 45.97), H (5.32 / 5.54), N (8.22 / 8.09).

Di-(1,3-diethyl-4-phenylimidazol-2-ylidene)gold(I)iodide **Au-23**

Chloro(1,3-diethyl-4-phenylimidazol-2-ylidene)gold(I) (95.6 mg, 0.22 mmol) was treated with 1 equivalent of 1,3-dimethyl-4-phenyl-1*H*-imidazol-2-iumiodide (74.2 mg, 0.23 mmol) and 1.5 equivalents of K_2CO_3 in a mixture of dichloromethane and methanol (1:1) for two days under vigorous stirring at room temperature. The obtained suspension was filtered over celite and the clear solution was evaporated under reduced pressure. The white resultant was dissolved in dichloromethane (2 mL) and then transferred into ethylacetate (30 mL) in an ultrasonic bath. The suspension was filtered to obtain the pure product. Yield: 78% white powder. ^{1}H NMR (500 MHz, $CDCl_3$): δ (ppm) = 7.54-7.46 (m, 6H; Ph-$H_{meta+para}$), 7.45-7.37 (m, 4H; Ph-H_{ortho}), 7.15 (s, 2H; ImH5), 4.43 (q, J = 7.3 Hz, 4H; CH_2), 4.28 (q, J = 7.3 Hz, 4H; CH_2), 1.63 (t, J = 7.3 Hz, 6H; CH_3), 1.43 (t, J = 7.2 Hz, 6H; CH_3); ^{13}C NMR (126 MHz, $CDCl_3$): δ (ppm) = 183.61 (Im-C2), 134.97 (Ph-$C1_{quat.}$), 129.73 (Ph-CH_{para}), 129.48 (Ph-CH_{ortho}), 129.14 (Ph-CH_{meta}), 127.43 (Im-$C4_{quat.}$), 119.00 (Im-C5), 47.08 (CH_2), 44.04 (CH_2), 17.57 (CH_3), 17.04 (CH_3); MS (ESI$^+$): m / z = 597.4 [M–I]$^+$; MS (ESI$^-$): m / z = 126.9 [I]$^-$; elemental analysis for $C_{26}H_{32}AuN_4I$ (%calc. / %found): C (43.11 / 43.20), H (4.45 / 4.41), N (7.73 / 7.67).

Di-(1,3-dimethyl-4-phenylimidazol-2-ylidene)gold(I)iodide **Au-24**

Chloro(1,3-dimethyl-4-phenylimidazol-2-ylidene)gold(I) (68.6 mg, 0.17 mmol) was treated with 1 equivalent of 1,3-dimethyl-4-phenyl-1*H*-imidazol-2-ium iodide (51.1 mg, 0.17 mmol) and 1.5 equivalents of K_2CO_3 in a mixture of dichloromethane and methanol (1:1) for two days under vigorous stirring at room temperature. The obtained suspension was filtered over celite and the clear solution was evaporated under reduced pressure. The white resultant was dissolved in dichloromethane (2 mL) and then transferred into diethyl ether (30 mL) in an ultrasonic bath. The suspension was filtered to obtain the pure product. Yield: 95% white powder. ^{1}H NMR (500 MHz, $CDCl_3$): δ (ppm) = 7.53-7.44 (m, 6H; Ph-$H_{meta+para}$), 7.44-7.36 (m, 4H; Ph-H_{ortho}), 7.09 (s, 2H; ImH5), 4.06 (d, J = 0.9 Hz, 6H; CH_3), 3.91 (d, J = 1.0 Hz, 6H; CH_3); ^{13}C NMR (126 MHz, $CDCl_3$): δ (ppm) = 185.34 (Im-C2), 135.67 (Ph-$C1_{quat.}$), 129.60 (Ph-CH_{para}), 129.32 (Ph-CH_{ortho}), 129.08 (Ph-CH_{meta}), 127.26 (Im-$C4_{quat.}$), 120.41 (Im-C5), 38.95 (CH_3), 36.91 (CH_3); MS (ESI$^+$): m / z = 541.3 [M–I]$^+$; MS (ESI$^-$): m / z = 126.9 [I]$^-$; elemental analysis for $C_{22}H_{24}AuN_4I$ (%calc. / %found): C (39.54 / 39.09), H (3.62 / 3.56), N (8.38 / 8.21).

Di-(1,3-dibenzyl-4-phenylimidazol-2-ylidene)gold(I) bromide **Au-28**

Chloro(1,3-dibenzyl-4-phenylimidazol-2-ylidene)gold(I) (78.5 mg, 0.14 mmol) was treated with 1 equivalent of 1,3-dibenzyl-4-phenyl-1*H*-imidazol-2-ium bromide (56.9 mg, 0.14 mmol) and

1.5 equivalents of K_2CO_3 in a mixture of dichloromethane and methanol (1:1) for two days under vigorous stirring at room temperature. The obtained suspension was filtered over celite and the clear solution was evaporated under reduced pressure. The white resultant was dissolved in dichloromethane (2 mL) and then transferred into diethyl ether (30 mL) in an ultrasonic bath. The suspension was filtered to obtain the pure product. Yield: 83% white powder. ^{1}H NMR (500 MHz, $CDCl_3$): δ (ppm) = 7.46-7.27 (m, 16H; ArH), 7.19 (s, 2H; ImH5), 7.19-7.12 (m, 10H; ArH), 6.95 – 6.87 (m, 4H; ArH), 5.39 (s, 4H; CH_2), 5.26 (s, 4H; CH_2); ^{13}C NMR (126 MHz, $CDCl_3$): δ (ppm) = 185.20 (Im-C2H), 136.5967 (Ph-$C1_{quat.}$), 136.13 (Benz-$C1_{quat.}$), 135.66 (Benz-$C1_{quat.}$), 129.74 (Ph-CH_{para}), 129.44 (Ph-CH_{ortho}), 129.02 (Ph-CH_{ortho}), 128.89 (Benz-CH_{ortho}), 128.81 (Benz-CH_{ortho}), 128.51 (Benz-CH_{para}), 127.93 (Benz-CH_{para}), 127.83 (Benz-CH_{meta}), 126.86 (Im-$C4_{quat.}$), 126.31 (Benz-CH_{meta}), 119.97 (Im-C5), 55.12 (CH_2), 51.83 (CH_2); MS (ESI^+): m / z = 854.4 $[M–Br]^+$, 325.2 $[NHC]^+$; elemental analysis for $C_{46}H_{40}AuN_4Br$ (%calc. / %found): C (59.68 / 59.89), H (4.36 / 4.40), N (6.05 / 5.99).

Di-(1,3-diisopropyl-4-phenylimidazol-2-ylidene)gold(I)iodide **Au-29**
Chloro(1,3-diisopropyl-4-phenylimidazol-2-ylidene)gold(I) (187.9 mg, 0.41 mmol) was treated with 1 equivalent of 1,3-Diisoproyl-4-phenyl-1*H*-imidazol-2-ium iodide (145.2 mg, 0.41 mmol) and 1.5 equivalents of K_2CO_3 in a mixture of dichloromethane and methanol (1:1) for two days under vigorous stirring at room temperature. The obtained suspension was filtered over celite and the clear solution was evaporated under reduced pressure. The white resultant was dissolved in dichloromethane (2 mL) and then transferred into diethyl ether (30 mL) in an ultrasonic bath. The suspension was filtered to obtain the pure product. Yield: 74% white powder. ^{1}H NMR (500 MHz, $CDCl_3$): δ (ppm) = 7.57-7.47 (m, 6H; Ph-$H_{meta+para}$), 7.42-7.34 (m, 4H; Ph-H_{ortho}), 7.14 (s, 2H; ImH5), 5.14 (hept, J = 6.8 Hz, 2H; CH), 4.60 (hept, J = 6.8 Hz, 2H; CH), 1.78 (d, J = 6.8 Hz, 12H; CH_3), 1.66 (d, J = 6.8 Hz, 12H; CH_3); ^{13}C NMR (126 MHz, $CDCl_3$): δ (ppm) = 179.85 (Im-C2), 135.67 (Ph-$C1_{quat.}$), 130.05 (Ph-CH_{ortho}), 129.93 (Ph-CH_{para}), 129.16 (Ph-CH_{meta}), 127.59 (Im-$C4_{quat.}$), 115.05 (Im-C5), 55.17 (CH), 50.13 (CH), 24.95 (CH_3), 23.81 (CH_3); MS (ESI^+): m / z = 653.5 $[M–I]^+$, 229.2 $[NHC]^+$; MS (ESI^-): m / z = 126.9 $[I]^-$; elemental analysis for $C_{30}H_{40}AuN_4I$ (%calc. / %found): C (46.16 / 46.11), H (5.17 / 5.25), N (7.18 / 7.12).

Synthesis of gold compounds Au-35 and Au-36

General for compounds Au-35 and Au-36

The zinc(II)-dithiocarbamato precursor $[Zn(SSC\text{-}Inp\text{-}GlcN1)_2]$ (Inp: isonipecotic moiety; GlcN1: 2-amino-2-deoxy-(α,β)-D-glucose) was synthesized as previously described. (A. Pettenuzzo et al., Dalton Trans. 2018, 47, 10721.) The gold(I)-carbene precursor $[AuCl(BImEt_2)]$ ($BImEt_2$:

1,3-diethylbenzimidazol-2-ylidene) was kindly provided by Prof. Ingo Ott (Technische Universität Braunschweig, Germany). [AuCl(PPh$_3$)] (STREM Chemicals), deuterated solvents for NMR analysis (Deutero) and other solvents were of reagent grade or comparable purity and were used as supplied without any further purification.

Melting points were recorded on a Stuart SMP10 digital melting point apparatus and are uncorrected. Elemental analyses (carbon, hydrogen and nitrogen) were performed with a Perkin Elmer 2400 CHNS/O Series II analyzer. FT-IR spectra were recorded from CsI disks at room temperature on a Perkin Elmer Frontier FT-MIR/FIR spectrophotometer in the range 4000-200 cm^{-1}. Data processing was carried out using OMNIC version 5.1 (Nicolet Instrument Corporation). NMR spectra were acquired in the specified deuterated solvent at room temperature on a Jeol 400 MHz spectrometer equipped with *z*-field gradients. ^{1}H and ^{13}C chemical shifts were referenced to TMS at 0.00 ppm *via* internal referencing to the residual peak of the deuterated solvent employed. ^{31}P chemical shifts were referenced to an external standard of 85% H_3PO_4 at 0 ppm. ^{1}H and ^{13}C{^{1}H} signals were assigned with the aid of [^{1}H,^{1}H] COSY, ^{13}C DEPT, [^{1}H,^{13}C] HSQC and [^{1}H,^{13}C] HMBC experiments. Data processing was carried out using MestReNova version 12.0 (Mestrelab Research S.L.).

[Au(BImEt$_2$)(SSC-Inp-GlcN1)] **Au-35**

A DMF solution (2 mL) of [Zn(SSC-Inp-GlcN1)$_2$] (49.4 mg, 0.06 mmol, 1 equiv.) was added dropwise under stirring to a DMF solution (2 mL) of [AuCl(BImEt$_2$)] (50.5 mg, 0.12 mmol, 2 equiv.) at room temperature, and the mixture was stirred for 5 minutes. Upon addition of diethyl ether (60 mL), the solution turned cloudy and a yellow solid residue formed. The precipitate was filtered off, washed with diethyl ether (3×10 mL), dichloromethane (3×10 mL), and then dried under vacuum over P_2O_5, yielding the title compound as a pale yellow solid (80.2 mg, 88%; solution α:β anomers ratio ≈ 2.5:1 based on the ^{1}H NMR spectrum).

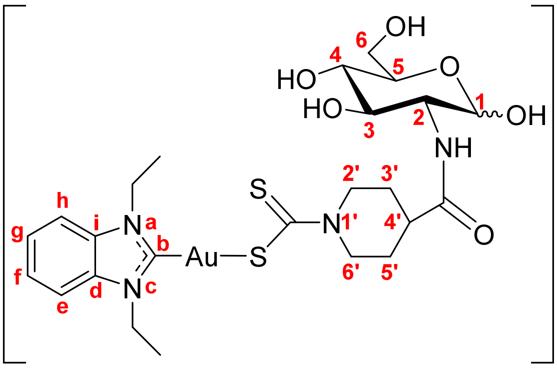

^{1}H NMR (400 MHz, DMSO-*d*$_6$, 25°C, TMS): δ (ppm) = 7.84-7.82 (m, 2.8H, C^{e,h}H), 7.69 (d, 0.4H, NH β), 7.65 (d, 1H, NH α), 7.48-7.46 (m, 2.8H, C^{f,g}H), 6.50 (d, 0.4H, C^{1}OH β), 6.42 (d, 1H, C^{1}OH α), 5.06 (br dt, 2.8H, C$^{2',6'}$H$_{eq}$), 4.91 (m, 1.4H, C^{1}H α + C^{4}OH β overlapped), 4.82 (m, 1.4H, C^{3}OH α and β overlapped), 4.70-4.50 (m, 5.4H, C^{4}OH α + C^{6}OH β + CH$_2$), 4.44 (m, 1.4H,

C^1H β + C^6OH α overlapped), 3.69-3.05 (m, 11.2H, C^2H + C^3H + C^4H + C^5H + C^6H_2 + $C^{2',6'}H_{ax}$ α and β overlapped), 2.58 (br tt, 1.4H, $C^{4'}H$), 1.82-1.71 (br m, 2.8H, $C^{3',5'}H_{eq}$), 1.65-1.54 (br m, 2.8H, $C^{3',5'}H_{ax}$), 1.42 (t, 8.4H, CH_3) ppm; ^{13}C NMR (100 MHz, DMSO-*d*$_6$, 25°C, TMS): δ (ppm) = 204.5 (NCSS), 184.2 (C^b), 173.9 (C=O), 132.4 ($C^{d,i}$), 124.2 ($C^{f,g}H$), 112.0 ($C^{e,h}H$), 95.4 (C^1H β), 90.5 (C^1H α), 76.9 (C^5H β), 74.2 (C^3H β), 72.1 (C^5H α), 71.1 (C^3H α), 70.8 (C^4H β), 70.4 (C^4H α), 61.2 (C^6H_2 β), 61.0 (C^6H_2 α), 57.0 (C^2H β), 54.3 (C^2H α), 50.5 ($C^{2',6'}H_2$), 43.1 (CH_2), 40.8 ($C^{4'}H$), 28.5 ($C^{3',5'}H_2$) ppm. No differentiation of the ^1H and ^{13}C signals of the carbene and the isonipecotic moieties due to the presence of both α and β anomers could be observed; IR (CsI): ṽ = 3401 (br m, O–H + N–H overlapped), 1657 (s, C=O (amide I)), 1543 (m, C–N–H (amide II)), 1483 (s, N–CSS), 1089/1059/1041 (m, C–OH), 998/914 (m, S=C–S), 566 (m, C–S), 506 (w, Au–C), 385 (m, Au–S) cm^{-1}; elemental analysis for $C_{24}H_{35}AuN_4O_6S_2$ (% calc. / % found): C 39.13 / 39.01, H 4.79 / 4.75, N 7.61 / 7.77; mp: 155-157°C (dec.).

[Au(SSC-Inp-GlcN1)(PPh_3)] **Au-36**:

A DMF solution (2 mL) of [Zn(SSC-Inp-GlcN1)$_2$] (60.1 mg, 0.07 mmol, 1 equiv.) was added dropwise under stirring to a DMF solution (2 mL) of [AuCl(PPh_3)] (74.8 mg, 0.15 mmol, 2.1 equiv.) at room temperature, and the mixture was stirred for 1 hour. Upon addition of diethyl ether (40 mL), the solution turned cloudy and an off-white solid residue formed. The precipitate was centrifuged and the bulk of supernatant discarded. The sticky residue was subsequently triturated with diethyl ether (3×10 mL), dichloromethane (3×10 mL), and then dried under vacuum over P_2O_5, yielding the title compound as a light yellow solid (85.3 mg, 68%, solution α:β anomers ratio ≈ 4:1 based on the ^1H NMR spectrum).

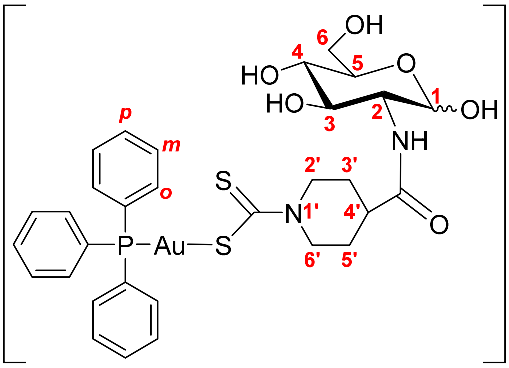

^1H NMR (400 MHz, DMSO-*d*$_6$, 25°C, TMS): δ (ppm) = 7.70 (d, 0.25H, NH β), 7.66 (d, 1H, NH α), 7.60-7.56 (m, 18.75H, ArH), 6.50 (d, 0.25H, C^1OH β), 6.42 (d, 1H, C^1OH α), 4.93-4.89 (m, 4.75H, C^1H α + C^3OH + $C^{2',6'}H_{eq}$ α and β overlapped), 4.61 (br d, 1.25H, C^4OH α and β overlapped), 4.54 (dd, 0.25H, C^6OH β), 4.44 (m, 1.25H, C^1H β + C^6OH α overlapped), 3.70-3.39 (m, 5.75H, C^2H α + C^4H α and β + C^5H α + C^6H_2 α and β overlapped), 3.29-3.20 (m, 3H, C^2H β + C^3H β + $C^{2',6'}H_{ax}$ overlapped), 3.14-3.03 (m, 1.25H, C^3H α + C^5H β overlapped), 2.58-2.45 (tt, 1.25H, $C^{4'}H$), 1.81-1.75 (br m, 2.5H, $C^{3',5'}H_{eq}$), 1.64-1.54 (br m, 2.5H, $C^{3',5'}H_{ax}$) ppm;

^{13}C NMR (100 MHz, DMSO-d_6, 25°C, TMS): δ (ppm) = 204.4 (NCSS), 173.9 (C=O), 133.7 (d, *o*-CH, $^2J_{C,P}$ = 15.7 Hz), 131.9 (d, *p*-CH, $^4J_{C,P}$ = 2.8 Hz), 129.8 (d, CP, $^1J_{C,P}$ = not detectable due to overlapping), 129.5 (d, *m*-CH, $^3J_{C,P}$ = 11.9 Hz), 95.4 (C^1H β), 90.5 (C^1H α), 76.9 (C^5H β), 74.2 (C^3H β), 72.1 (C^5H α), 71.1 (C^3H α), 70.9 (C^4H β), 70.4 (C^4H α), 61.1 (C^6H_2 β), 61.0 (C^6H_2 α), 57.0 (C^2H β), 54.3 (C^2H α), 50.7 ($C^{2',6'}H_2$), 41.0 ($C^{4'}H$), 28.5 ($C^{3',5'}H_2$) ppm. No differentiation of the ^1H and ^{13}C signals of the triphenylphosphine and the isonipecotic moieties due to the presence of both α and β anomers could be observed. ^{31}P NMR (162 MHz, DMSO-d_6, 25°C, H_3PO_4): δ (ppm) = 36.5 ppm. IR (CsI): ṽ = 3401 (br m, O–H + N–H overlapped), 1650 (s, C=O (amide I)), 1544 (m, C–N–H (amide II)), 1481 (s, N–CSS), 1100 (s, P–Ph_3), 1059 (m, C–OH), 998/913 (m, S=C–S), 710/693 (s, P–Ph_3), 578 (m, C–S), 539/509/426 (m, P–Ph_3), 369 (m, Au–S), 272/241 (m, P–Ph_3 + Au–P) cm^{-1}; elemental analysis for $C_{31}H_{36}AuN_2O_6PS_2$ (% calc. / % found): C 45.15/44.99, H 4.40/4.39, N 3.40/3.36; mp: 170-172°C (dec.).

2) Synthesis of ruthenium compounds

General for compounds Ru-2, Ru-3 and Ru-9: Same as for **Au-6 - Au-29**

General method for Ru-2, Ru-3 and Ru-9

The starting benzimidazolium bromides were dissolved in dichloromethane, 0.6 equivalents of silver oxide were added and the mixture was stirred for 12 h at room temperature. The Ru(II)-dimer [(*p*-Cym)RuX_2]$_2$ (0.5 equivalents) was added and stirring was continued for 2 days at room temperature. The progress of the reaction was monitored by thin-layer chromatography (eluent: 5% methanol in dichloromethane). Precipitated silver halogenide was removed by filtration over celite, the volume of the filtrate was reduced under vacuum. Additional purification was done by column chromatography on silica (eluent: 5% methanol in dichloromethane), evaporation, and recrystallisation (dichloromethane / n-hexane).

(1,3-Dibenzyl-5-methyl-1*H*-benzimidazol-2-ylidene)-dibromido-(η^6-*p*-cymene)ruthenium(II)
Ru-2

synthesis starting from: 1,3-dibenzyl-5-methyl-1*H*-benzimidazolium bromide (89.1 mg, 0.23 mmol); yield: 47.5 mg (0.07 mmol, 29.6 %), dark red powder; ^1H-NMR (600 MHz, $CDCl_3$-d_1) δ = 7.35 (m, Bn^1/Bn^2-H3-H5, 4H), 7.31 – 7.28 (m, Bn^1/Bn^2-H3-H5, 2H), 7.14 – 7.11 (m, Bn^1/Bn^2-H2+H6, 4H), 6.90 – 6.85 (m, BeIm-H6+H7, 2H), 6.82 (dd, $^4J_{H,H}$ = 1.4 Hz, $^5J_{H,H}$ = 0.7 Hz, BeIm-H4, 1H), 6.56 (d, $^2J_{H,H}$ = 16.9 Hz, Bn^2-CH_2, 1H), 6.45 (d, $^2J_{H,H}$ = 16.9 Hz, Bn^1-CH_2,1H), 5.95 (d, $^2J_{H,H}$ = 16.9 Hz, Bn^1-CH_2, 1H), 5.86 (d, $^2J_{H,H}$ = 16.9 Hz, Bn^2-CH_2, 1H), 5.38 (d, $^3J_{H,H}$ = 8.5 Hz, *p*-Cym-H3+H5, 2H), 5.00 (d, $^3J_{H,H}$ = 8.5 Hz, *p*-Cym-H2+H6, 2H), 2.99 (hept, $^3J_{H,H}$ = 7.0 Hz, 1H), 2.29 (s, BeIm-CH_3, 3H), 1.89 (s, *p*-Cym-CH_3, 3H), 1.19 (d, $^3J_{H,H}$ = 7.0 Hz, *p*-Cym-CH-CH_3, 6H);

^{13}C-NMR (151 MHz, $CDCl_3$-d_1) δ = 189.61 (BeIm-C2$_{quart.}$), 137.51 (Bn^2-C1$_{quart.}$), 137.39 (Bn^1-C1$_{quart.}$), 135.95 (BeIm-C7a$_{quart.}$), 133.80 (BeIm-C3a$_{quart.}$), 133.27 (BeIm-C5$_{quart.}$), 128.89 (Bn^1/Bn^2-C3-C5), 128.86 (Bn^1/Bn^2-C3-C5), 128.80 (Bn^1/Bn^2-C3-C5), 127.44 (Bn^1/Bn^2-C3-C5), 126.20 (Bn^1/Bn^2-C2+C6), 126.03 (Bn^1/Bn^2-C2+C6), 124.42 (BeIm-C6), 111.58 (BeIm-C4), 111.36 (BeIm-C7), 109.48 (*p*-Cym-C4$_{quart.}$), 97.62 (*p*-Cym-C1$_{quart.}$), 85.96 (*p*-Cym-C3+C5), 85.79 (*p*-Cym-C3+C5), 83.55 (*p*-Cym-C2+C6), 54.59 (Bn^1-CH_2), 54.18 (Bn^2-CH_2), 30.80 (*p*-Cym-CH), 22.63 (*p*-Cym-CH-CH_3), 22.42 (*p*-Cym-CH-CH_3), 21.41 (BeIm-CH_3), 18.75 (*p*-Cym-CH_3); elemental analysis for $C_{32}H_{34}Br_2N_2Ru$ (theor./found [%]): C (54.32/54.18), H (4.84/4.78) N (3.96/4.12); MS (ESI): m/z 803.40 [M-Br+M-2Br-*p*-Cym-Ru]$^+$, 629.09 [M-Br]$^+$, 549.18 [M-2Br]$^+$, 428.94 [M-2Br-*p*-Cym]$^+$.

(1,3-Dibenzyl-5-methyl-1*H*-benzimidazol-2-ylidene)-diiodido-(η^6-*p*-cymol)-ruthenium(II) **Ru-3** synthesis starting from: 1,3-Dibenzyl-5-methyl-1*H*-benzimidazolium iodide (121.9 mg, 0.28 mmol); yield: 60.1 mg (0.07 mmol, 27.1 % dark red powder; ^{1}H-NMR (500 MHz, $CDCl_3$-d_1) δ = 7.37 – 7.28 (m, Bn^1/Bn^2-H3-H5, 6H), 7.12 – 7.08 (m, Bn^1/Bn^2-H2+H6, 4H), 6.85 (ddd, $^3J_{H,H}$ = 8.3 Hz, $^4J_{H,H}$ = 1.4 Hz, BeIm-H6, 1H), 6.82 (dd, $^3J_{H,H}$ = 8.3 Hz, $^5J_{H,H}$ = 0.7 Hz, BeIm-H7, 1H), 6.77 (dt, $^4J_{H,H}$ = 1.4 Hz, $^5J_{H,H}$ = 0.7 Hz, BeIm-H4, 1H), 6.50 (d, $^2J_{H,H}$ = 17.0 Hz, Bn^2-CH_2, 1H), 6.40 (d, $^2J_{H,H}$ = 16.7 Hz, Bn^1-CH_2, 1H), 6.07 (d, $^2J_{H,H}$ = 16.7 Hz, Bn^1-CH_2,1H), 5.97 (d, $^2J_{H,H}$ = 17.0 Hz, Bn^2-CH_2, 1H), 5.48 (d, *J* = 5.8 Hz, *p*-Cym-H3+H5, 2H), 5.08 (d, *J* = 21.2 Hz, *p*-Cym-H2+H6, 2H),3.27 (hept, *J* = 6.9 Hz, *p*-Cym-CH, 1H), 2.28 (s, BeIm-CH_3, 3H), 1.96 (s, *p*-Cym-CH_3, 3H), 1.18 (d, *J* = 6.9 Hz, *p*-Cym-CH-CH_3, 6H); ^{13}C-NMR (126 MHz, $CDCl_3$-d_1) δ = 188.74 (BeIm-C2$_{quart.}$), 137.13 (Bn2-C1$_{quart.}$), 137.03 (Bn1-C1$_{quart.}$), 135.90 (BeIm-C7a$_{quart.}$), 133.77 (BeIm-C3a$_{quart.}$), 133.24 (BeIm-C5$_{quart.}$), 128.81 (Bn^1/Bn^2-C3-C5), 128.75 (Bn^1/Bn^2-C3-C5), 127.48 (Bn^1/Bn^2-C3-C5), 126.35 (Bn^1/Bn^2-C2+C6), 126.17 (Bn^1/Bn^2-C2+C6), 124.36 (BeIm-C6), 111.74 (BeIm-C4), 111.50 (BeIm-C7) , 110.68 (*p*-Cym-C4$_{quart.}$), 98.52 (*p*-Cym-C1$_{quart.}$), 85.93 (*p*-Cym-C3+C5), 84.05 (*p*-Cym-C2+C6), 57.55 (Bn^1-CH_2), 57.17 (Bn^2-CH_2), 31.60 (*p*-Cym-CH), 22.92 (*p*-Cym-CH-CH_3), 22.63 (*p*-Cym-CH-CH_3), 21.39 (BeIm-CH_3), 19.29 (*p*-Cym-CH_3); elemental analysis for $C_{32}H_{34}I_2N_2Ru$ (theor./found [%]): C (47.95/47.69), H (4.28/4.40) N (3.50/3.25); MS (ESI): m/z 1477.07 [M-I+M]$^+$, 675.08 [M-I+H]$^+$, 413.05 [M-2I-*p*-Cym-Ru]$^+$

(1,3-Dibenzyl-1*H*-imidazol-2-ylidene)-dichlorido-(η^6-*p*-cymol)-ruthenium(II) **Ru-9**
synthesis starting from: 1,3-Dibenzyl-1*H*-imidazolium bromide (99.5 mg, 0.30 mmol); yield: 113.6 mg (0.20 mmol, 67.8 %), orange-red powder; ^{1}H-NMR (600 MHz, $CDCl_3$-d_1) δ = 7.41 – 7.35 (m, Bn^1+Bn^2-H2+H6, 4H), 7.35 – 7.28 (m, Bn^1+Bn^2-H3-H5, 6H), 6.82 (d, $J_{H,H}$ = 1.7 Hz, Im-H4+H5, 2H), 5.85 (s, Bn^1+Bn^2-CH_2, 2H), 5.67 (s, Bn^1+Bn^2-CH_2, 2H), 5.39 – 5.33 (m, *p*-Cym-H3+H5, 2H), 5.07 – 5.02 (m, *p*-Cym-H2+H6, 2H), 2.87 (hept, $^3J_{H,H}$ = 7.1 Hz, *p*-Cym-CH, 1H), 2.03 (s, *p*-Cym-CH_3, 3H), 1.23 (dd, $^3J_{H,H}$ = 7.0, 1.7 Hz, *p*-Cym-CH-CH_3, 6H); ^{13}C-NMR (151

MHz, $CDCl_3$-d_1) δ = 175.55 (Im-C2), 137.41 (Bn^1+Bn^2-$C1_{quart.}$), 128.84 (Bn^1+Bn^2-C2+C6) , 127.96 (Bn^1+Bn^2-C3-C5), 127.81 (Bn^1+Bn^2-C3-C5), 122.90 (BeIm-C4+C5), 107.86 (*p*-Cym-$C4_{quart.}$) , 97.81 (*p*-Cym-$C1_{quart.}$), 85.06 (*p*-Cym-C2+C6), 83.30 (*p*-Cym-C3+C5), 55.03 (Bn^1+Bn^2-CH_2), 30.72 (*p*-Cym-CH), 22.57 (*p*-Cym-CH-CH_3), 18.69 (*p*-Cym-CH_3); elemental analysis for $C_{27}H_{30}Cl_2N_2Ru$ (theor./found [%]): C (58.48/57.67), H (5.45/5.21) N (5.05/4.85); MS (ESI): m/z 1073.20 $[M+M-Cl]^+$, 519.11 $[M-Cl]^+$, 349.03 [M-2Cl-*p*-Cym-H]$^+$

General for Ru-17

NMR were run on Bruker Avance III 600 spectrometer; Mass spectra were recorded using EI method on a Finnigan MAT 95 mass spectrometer and ESI on a Synapt G2-Si mass spectrometer (Waters); IR spectra were recorded on an FTIR Nexus Nicolet apparatus; Microanalyses were performed by the Analytical Services of the Polish Academy of the Sciences (Łódź).

3-(1-thyminyl)-1-(1-ruthenocenyl)-prop-1-ene **Ru-17**:

Ytterbium(III) trifluoromethanesulfonate (15 mg, 0.024 mmol) was added to solution of 3-(1-thyminyl)-1-(1-ruthenocenyl)-propan-1-ol (100 mg, 0.24 mmol) in ethylene glycol (30 mL) at ambient temperature. The reaction mixture was stirred at 110 ^{0}C for 20 h. Then, the reaction was cool to ambient temperature, poured into water and the obtained mixture was extracted with chloroform. The organic layer was separated, dried over anhydrous Na_2SO_4, filtered and all volatiles were evaporated under reduced pressure. The remaining material was subjected to column chromatography on silica gel with dichloromethane / methanol (50 : 1 v/v) as eluent. Crystallization from dichloromethane / *n*-hexane gave product. Yield: 58 % (55 mg), colorless solid. ^{1}H NMR (600 MHz, DMSO-d_6): δ (ppm) = 11.25 (s, 1H, NH), 7.49 (d, 1H, J = 0.6 Hz, H6 thymine), 6.18 (d, 1H, J = 15.6 Hz, vinyl), 5.76 (dt, 1H, J = 15.6 Hz, 6.0 Hz, vinyl), 4.84 (pt, 2H, J = 1.8 Hz, C_5H_4), 4.57 (pt, 2H, J = 1.8 Hz, C_5H_4), 4.49 (s, 5H, C_5H_5), 4.19 (d, 2H, J = 6.0 Hz, CH_2), 1.75 (d, 3H, J = 0.6 Hz, CH_3 thymine). ^{13}C NMR (150 MHz, DMSO-d_6): δ (ppm) = 164.4, 150.8, 141.0, 130.2, 120.7, 108.8, 85.8, 71.2, 70.9, 69.4, 48.6, 12.0; MS (ESI$^+$): m/z = 397.0505 $[M+H]^+$ (calcd for $C_{18}H_{19}N_2O_2Ru$: 397.0490); FTIR (KBr ν [cm^{-1}]): 3095, 3042, 1698, 1680, 1654, 1453, 1333, 1215, 962, 808; elemental analysis for $C_{18}H_{18}N_2O_2Ru$ + ½ *n*-hexane (%calc./%found): C (57.52 / 57.27), H (5.75 / 6.07), N (6.39 / 6.08).

3) Synthesis of rhodium compounds Rh-1 - Rh11

General for compounds Rh-1 -Rh-11: Same as for **Au-6 - Au-29**

(Chlorido)(cycloocta-1,5-dien)(1,3-diethyl-4-(phenylimidazol-2-yliden))rhodium(I) **Rh-1**:

1,3-Diethyl-(4-phenyl-1H-imidazol-2-ium)iodide (0.1 g 0.30 mmol) was treated with 0.5 equivalents of Ag_2O (0.035 g, 0.15 mmol) for 4 h in 10 mL of dichloromethane under vigorous stirring at room temperature in the dark. After this period chlorido(cycloocta-1,5-dien)rhodium)dimer (0.075 g, 0.15 mmol) was added and the reaction was stirred 18 h. The obtained suspension was filtered over celite and the solvent was evaporated under reduced pressure to give an oil. The resulting oil is raised on silica gel and purified by column chromatography on silica gel (EtOAc/n-hexane: 1/1). The isolated solid is dried in the vacuum drying gun at 40 °C. Yield: 96% yellow crystalline solid. ^{1}H NMR (600 MHz, $CDCl_3$): δ (ppm) = 7.46-7.37 (m, 3H, $C_{3',4',5'}$, CH), 7.35-7.31 (m, 2H, $C_{2',6'}$, CH), 6.80 (s, 1H, C_5, CH), 5.06-5.00 (m, 2H, cis-CH-COD, CH), 4.85 (dq, $^2J_{H,H}$ = 13.5 Hz, $^3J_{H,H}$ = 7.3 Hz, 1H, $N_{3\text{-Ethyl}}$, CH_2), 4.72 (dq, $^2J_{H,H}$ = 13.4 Hz, $^3J_{H,H}$ = 7.4 Hz, 1H, $N_{1\text{-Ethyl}}$, CH_2), 4.61-4.55 (m, 1H, $N_{1\text{-Ethyl}}$, CH_2), 4.55-4.49 (m, 1H, $N_{3\text{-Ethyl}}$, CH_2), 3.41-3.33 (m, 2H, trans-CH-COD, CH), 2.51-2.34 (m, 4H, CH_2-COD, $CH_{2,equatorial}$), 2.01-1.90 (m, 4H, CH_2-COD, $CH_{2,axial}$), 1.56 (t, $^3J_{H,H}$ = 7.4 Hz, 3H, $N_{3\text{-Ethyl}}$, CH_3), 1.27 (t, $^3J_{H,H}$ = 7.2 Hz, 3H, $N_{1\text{-Ethyl}}$, CH_3); ^{13}C NMR (151 MHz, $CDCl_3$) δ (ppm) = 182.64 (d, $^1J_{Rh,C}$ = 51.0 Hz, 1C, C_2, C-Rh), 134.73 (s, 1C, C_4, C), 129.13 (s, 1C, $C_{1'}$, C), 128.81 – 128.73 (m, 5C, $C_{2',3',4',5',6'}$, CH), 118.14 (s, 1C, C_5, CH), 98.25 (dd, $^1J_{Rh,C}$ = 6.9 Hz, $^2J_{Rh,C}$ = 5.3 Hz, 2C, cis-CH-COD, CH), 68.12 (dd, $^1J_{Rh,C}$ = 54.4 Hz, $^2J_{Rh,C}$ = 14.7 Hz, 2C, trans-CH-COD, CH), 45.82 (s, 1C, $N_{1\text{-Ethyl,}}$ CH_2), 43.87 (s, 1C, $N_{3\text{-Ethyl,}}$ CH_2), 32.95 (d, $^3J_{Rh,C}$ = 59.1 Hz, 2C, CH_2-COD, CH_2), 28.89 (d, $^3J_{Rh,C}$ = 50.1 Hz, 2C, CH_2-COD, CH_2), 16.32 (s, 1C, N_3-Ethyl, CH_3), 16.22 (s, 1C, N_1-Ethyl, CH_3); MS (ESI$^+$): m/z: 446,0 [M]$^+$; elemental analysis for $C_{21}H_{28}ClN_2Rh$ (%calc./%found): C (56.45 / 56.42), H (6.32 / 6.34), N (6.27 / 6.12).

(Bromido)(cycloocta-1,5-dien)(1,3-diethyl-4-(phenylimidazol-2-yliden))rhodium(I) **Rh-2**:
1,3-Diethyl-(4-phenyl-1H-imidazol-2-ium)iodid (0.1 g 0.30 mmol) was treated with 0.5 equivalents of Ag_2O (0.035 g, 0.15 mmol) for 4 h in 10 mL of dichloromethane under vigorous stirring at room temperature in the dark. After this period bromido(cycloocta-1,5-dien)rhodium)dimer (0.089 g, 0.15 mmol) was added and the reaction was stirred 18 h. The obtained suspension was filtered over celite and the solvent was evaporated under reduced pressure to give an oil. The resulting oil is raised on silica gel and purified by column chromatography on silica gel (EtOAc/n-hexane: 1/1). The isolated solid is dried in the vacuum drying gun at 40 °C. Yield 86% yellow crystalline solid. ^{1}H NMR (400 MHz, $CDCl_3$) δ (ppm) = 7.45-7.38 (m, 3H, $C_{3',4',5'}$, CH), 7.36-7.31 (m, 2H, $C_{2',6'}$, CH), 6.81 (s, 1H, C_5, CH), 5.15-5.07 (m, 2H, cis-CH-COD, CH), 4.87 (dq, $^2J_{H,H}$ = 13.7 Hz, $^3J_{H,H}$ = 7.3 Hz, 1H, $N_{3\text{-Ethyl,}}$ CH_2), 4.70 (dq, $^2J_{H,H}$ = 13.4 Hz, $^3J_{H,H}$ = 7.4 Hz, 1H, $N_{1\text{-Ethyl}}$, CH_2), 4.52 (ddq, $^2J_{H,H}$ = 13.4 Hz, $^3J_{H,H}$ = 7.2 Hz, 2H, 1H, $N_{1\text{-Ethyl}}$ + 1H, $N_{3\text{-Ethyl}}$, CH_2), 3.46-3.36 (m, 2H, trans-CH-COD, CH), 2.50 – 2.30 (m, 4H, CH_2-COD, $CH_{2,equatorial}$), 2.01-1.85 (m, 4H, CH_2-COD, $CH_{2,axial}$), 1.55 (t, $^3J_{H,H}$ = 7.3 Hz, 3H, $N_{1\text{-Ethyl}}$, CH_3), 1.23 (t, $^3J_{H,H}$ = 7.2 Hz, 3H, $N_{3\text{-Ethyl}}$, CH_3). ^{13}C NMR (101 MHz, $CDCl_3$) δ (ppm) = 182.46

(d, $^1J_{Rh,C}$ = 50.2 Hz, 1C, C_2, C-Rh), 134.82-134.77 (m, 1C, C_4, C), 129.18 (s, 1C, $C_{1'}$, C), 128.82-128.68 (m, 5C, $C_{2',3',4',5',6'}$, CH), 118.24 (s, 1C, C_5, CH), 97.67 (dd, $^1J_{Rh,C}$ = 6.8 Hz, $^2J_{Rh,C}$ = 1.6 Hz, 2C, cis-CH-COD, CH), 69.15 (dd, $^1J_{Rh,C}$ = 25.3 Hz, $^2J_{Rh,C}$ = 14.6 Hz, 2C, trans-CH-COD, CH), 45.78 (s, 1C, $N_{1\text{-}Ethyl}$, CH_2), 43.90 (s, 1C, $N_{3\text{-}Ethyl}$, CH_2), 32.75 (d, $^3J_{Rh,C}$ = 40.4 Hz, 2C, CH_2-COD, CH_2), 29.13 (d, $^3J_{Rh,C}$ = 34.2 Hz, 2C, CH_2-COD, CH_2), 16.05 (s, 1C, N_3-Ethyl, CH_3), 16.01 (s, 1C, N_1-Ethyl, CH_3); MS (ESI^+): m/z: 490.0 $[M]^+$; elemental analysis for $C_{21}H_{28}BrN_2Rh$ (%calc./%found): C (51.34 / 51.71), H (5.75 / 5.81), N (5.70 / 5.53).

(Iodido)(cycloocta-1,5-dien)(1,3-diethyl-4-(phenylimidazol-2-yliden))rhodium(I) **Rh-3**

Rh-1 (0.05 g) was dissolved in acetone (2.0 mL) in a microwave tube with a stirring containing 2.0 eq. potassium iodide (0.037 g) and stirred for 30 min at 20 watts, 11 psi and 56 °C. The mixture was transferred to a round bottom flask and the solvent was removed under vacuo. The resulting substance was purified by column chromatography on silica gel (DCM). The isolated solid was dried in the drying gun in vacuum at 40 °C. Yield 89 % yellow crystalline solid. ^{1}H NMR (500 MHz, $CDCl_3$) δ (ppm) = 7.45-7.38 (m, 3H, $C_{3',4',5'}$, CH), 7.36-7.33 (m, 2H, $C_{2',6'}$, CH), 6.83 (s, 1H, C_5, CH), 5.26-5.20 (m, 2H, cis-CH-COD, CH), 4.85 (dq, $^2J_{H,H}$ = 13.5 Hz, $^3J_{H,H}$ = 7.3 Hz, 1H, $N_{3\text{-}Ethyl}$, CH_2), 4.65 (dq, $^2J_{H,H}$ = 13.4 Hz, $^3J_{H,H}$ =7.4 Hz, 1H, $N_{1\text{-}Ethyl}$, CH_2), 4.54-4.43 (m, 2H, 1H, $N_{1\text{-}Ethyl}$ + 1H, $N_{3\text{-}Ethyl}$, CH_2), 3.55-3.48 (m, 2H, trans-CH-COD, CH), 2.41-2.29 (m, 4H, CH_2-COD, $CH_{2,equatorial}$), 2.03-1.91 (m, 2H, CH_2-COD, $CH_{2,axial,cis}$), 1.87-1.78 (m, 2H, CH_2-COD, $CH_{2,axial,trans}$), 1.53 (t, $^3J_{H,H}$ = 7.3 Hz, 3H, $N_{1\text{-}Ethyl}$, CH_3), 1.19 (t, $^3J_{H,H}$ = 7.2 Hz, 3H, $N_{3\text{-}Ethyl}$, CH_3); ^{13}C NMR (126 MHz, $CDCl_3$) δ (ppm) = 181.93 (d, $^1J_{Rh,C}$ = 48.8 Hz, C_2, C-Rh), 134.89 (s, 1C, C_4, C), 129.23 (s, 1C, $C_{1'}$, C), 128.83-128.54 (m, 4C, $C_{2',3',5',6'}$, CH), 118.35 (s, 1C, C_5, CH), 96.17 (dd, $^1J_{Rh,C}$ = 6.8 Hz, $^2J_{Rh,C}$ = 4.7 Hz, 2C, cis-CH-COD, CH), 71.67 (t, $^2J_{Rh,C}$ = 13.8 Hz, 2C, trans-CH-COD, CH), 45.70 (s, 1C, $N_{1\text{-}Ethyl}$, CH_2), 43.89 (s, 1C, $N_{3\text{-}Ethyl}$, CH_2), 32.34 (d, $^1J_{Rh,C}$ = 56.3 Hz, 2C, CH_2-COD, CH_2), 29.58 (d, $^1J_{Rh,C}$ = 48.0 Hz, 2C, CH_2-COD, CH_2), 15.62 (s, 1C, N_3-Ethyl, CH_3), 15.50 (s, 1C, N_1-Ethyl, CH_3); MS (ESI^+)): m/z: 537.9 $[M]^+$; elemental analysis for $C_{21}H_{28}IN_2Rh$ (%calc / %found): C (46.86 / 47.20), H (5.24 / 5.30), N (5.20 / 4.95).

(Chlorido)(cycloocta-1,5-dien)(1,3-diethyl-4-(4'-fluorphenylimidazol-2-yliden)) rhodium(I) **Rh-4**

1,3-Diethyl-(4-(4'-Fluorphenyl)-1H-imidazol-2-ium)iodid (0.1 g 0.29 mmol) was treated with 0.5 equivalents of Ag_2O (0.034 g, 0.14 mmol) for 4 h in 10 mL of dichloromethane under vigorous stirring at room temperature in the dark. After this period chlorido(cycloocta-1,5-dien)rhodium)dimer (0.071 g, 0.14 mmol) was added and the reaction was stirred 18 h. The obtained suspension was filtered over celite and the solvent was evaporated under reduced pressure to give an oil. The resulting oil is raised on silica gel and purified by column chromatography on silica gel (EtOAc/n-hexane: 1/1). The isolated solid is dried in the vacuum drying gun at 40 °C. Yield: 94% yellow crystalline solid. ^{1}H NMR (600 MHz, $CDCl_3$): δ (ppm) =

7.32-7.28 (m, 2H, $C_{2',6'}$, CH), 7.15-7.10 (m, 2H, $C_{3',5'}$, CH), 6.79 (s, 1H, C_5, CH), 5.06-5.00 (m, 2H, cis-CH-COD, CH), 4.82 (dq, $^2J_{H,H}$ = 13.8 Hz, $^3J_{H,H}$ = 7.3 Hz, 1H, $N_{3\text{-Ethyl}}$, CH_2), 4.71 (dq, $^2J_{H,H}$ = 13.6 Hz, $^3J_{H,H}$ = 7.2 Hz, 1H, $N_{1\text{-Ethyl}}$, CH_2), 4.57 (dq, $^2J_{H,H}$ = 13.6 Hz, $^3J_{H,H}$ = 7.2 Hz, 1H, $N_{1\text{-Ethyl}}$, CH_2), 4.48 (dq, $^2J_{H,H}$ = 13.9 Hz, $^3J_{H,H}$ = 7.3 Hz, 1H, $N_{3\text{-Ethyl}}$, CH_2), 3.40 – 3.31 (m, 2H, trans-CH-COD, CH), 2.50 – 2.34 (m, 4H, CH_2-COD, $CH_{2,equatorial}$), 2.01 – 1.91 (m, 4H, CH_2-COD, $CH_{2,axial}$), 1.55 (t, $^3J_{H,H}$ = 7.3 Hz, 3H, $N_{1\text{-Ethyl}}$, CH_3), 1.27 (t, $^3J_{H,H}$ = 7.2 Hz, 3H, $N_{3\text{-Ethyl}}$, CH_3); ^{13}C NMR (151 MHz, $CDCl_3$): δ (ppm) = 182.88 (d, $^1J_{Rh,C}$ = 51.1 Hz, 1C, C_2, C-Rh), 162.95 (d, $^1J_{C,F}$ = 249.3 Hz, 1C, $C_{4'}$, C-F), 133.65 (s, 1C, C_4, C), 130.76 (d, $^3J_{C,F}$ = 8.3 Hz, 2C, $C_{2',6'}$, CH), 125.15 (d, $^4J_{C,F}$ = 3.5 Hz, 1C, $C_{1'}$, C), 118.29 (s, 1C, C_5, CH), 115.93 (d, $^2J_{C,F}$ = 21.8 Hz, 2C, $C_{3',5'}$, CH), 98.36 (t, $^2J_{Rh,C}$ = 6.4 Hz, 2C, cis-CH-COD, CH), 68.14 (dd, $^1J_{Rh,C}$ = 55.0 Hz, $^2J_{Rh,C}$ = 14.6 Hz, 2C, trans-CH-COD, CH), 45.86 (s, 1C, $N_{1\text{-Ethyl}}$, CH_2), 43.82 (s, 1C, $N_{3\text{-Ethyl}}$, CH_2), 32.94 (d, $^1J_{Rh,C}$ = 57.0 Hz, 2C, CH_2-COD, CH_2), 28.88 (d, $^1J_{Rh,C}$ = 48.7 Hz, 2C, CH_2-COD, CH_2), 16.31 (s, 1C, N_3-Ethyl, CH_3), 16.20 (s, 1C, N_1-Ethyl, CH_3) 16.26 (d, $^4J_{Rh,C}$ = 16.6 Hz, 2C, $N_{1,3\text{-Ethyl}}$, CH_3);^{19}F NMR (377 MHz, $CDCl_3$): δ (ppm) = -112.44 (tt, $^3J_{F,H}$ = 8.4 Hz, $^4J_{F,H}$ = 5.2 Hz); MS (ESI^+): m/z: 464.0 $[M]^+$; elemental analysis for $C_{21}H_{27}ClFN_2Rh$ (%calc. / %found): (54.26 / 54.63), H (5.86 / 5.83), N (6.03 / 5.87).

(Bromido)(cycloocta-1,5-dien)(1,3-diethyl-4-(4'-fluorphenylimidazol-2-yliden)) rhodium(I) **Rh-5** 1,3-Diethyl-(4-(4'-Fluorphenyl)-1H-imidazol-2-ium)iodid (0.061 g 0.18 mmol) was treated with 0.5 equivalents of Ag_2O (0.021 g, 0.091 mmol) for 4 h in 10 mL of dichloromethane under vigorous stirring at room temperature in the dark. After this period bromido(cycloocta-1,5-dien)rhodium)dimer (0.052 g, 0.091 mmol) was added and the reaction was stirred 18 h. The obtained suspension was filtered over celite and the solvent was evaporated under reduced pressure to give an oil. The resulting oil is raised on silica gel and purified by column chromatography on silica gel (EtOAc/n-hexane: 1/1). The isolated solid is dried in the vacuum drying gun at 40 °C. Yield: 79 % yellow crystalline solid. ^1H NMR (500 MHz, $CDCl_3$): δ (ppm) = 7.33-7.29 (m, 2H, $C_{2',6'}$, CH), 7.15-7.10 (m, 2H, $C_{3',5'}$, CH), 6.80 (s, 1H, C_5, CH), 5.14-5.08 (m, 2H, cis-CH-COD, CH), 4.83 (dq, $^2J_{H,H}$ = 13.7 Hz, $^3J_{H,H}$ = 7.2 Hz, 1H, $N_{3\text{-Ethyl}}$, CH_2), 4.69 (dq, $^2J_{H,H}$ = 13.4, $^3J_{H,H}$ = 7.4 Hz, 1H, $N_{1\text{-Ethyl}}$, CH_2), 4.54 (dq, $^2J_{H,H}$ = 13.4 Hz, $^3J_{H,H}$ = 7.4 Hz, 1H, $N_{1\text{-Ethyl}}$, CH_2), 4.47 (dq, $^2J_{H,H}$ = 13.7 Hz, $^3J_{H,H}$ = 7.1 Hz, 1H, $N_{3\text{-Ethyl}}$, CH_2), 3.45 – 3.36 (m, 2H, trans-CH-COD, CH), 2.47-2.32 (m, 4H, CH_2-COD, $CH_{2, equatorial}$), 2.01-1.83 (m, 4H, CH_2-COD, $CH_{2, axial}$), 1.54 (t, $^3J_{H,H}$ = 7.4 Hz, 3H, $N_{1\text{-Ethyl}}$, CH_3), 1.24 (t, $^3J_{H,H}$ = 7.2 Hz, 3H, $N_{3\text{-Ethyl}}$, CH_3); ^{13}C NMR (126 MHz, $CDCl_3$): δ (ppm) = 182.64 (d, $J_{Rh,C}$ = 50.1 Hz, 1C, C_2, C-Rh), 162.93 (d, $J_{C,F}$ = 249.3 Hz, 1C, $C_{4'}$, C-F), 133.70 (s, 1C, C_4, C), 130.69 (d, $^3J_{C,F}$ = 8.3 Hz, 2C, $C_{2',6'}$, CH), 125.18 (d, $^4J_{C,F}$ = 3.4 Hz, 1C, $C_{1'}$, C), 118.37 (s, 1C, C_5, CH), 115.94 (d, $^2J_{C,F}$ = 21.7 Hz, 2C, $C_{3',5'}$, CH), 97.76 (dd, $^1J_{Rh,C}$= 6.8 Hz, $^2J_{Rh,C}$ = 2.7 Hz, 2C, cis-CH-COD, CH), 69.18 (dd, $^1J_{Rh,C}$ = 33.3 Hz, $^2J_{Rh,C}$ = 14.6 Hz, 2C, trans-CH-COD, CH), 45.80 (s, 1C, $N_{1\text{-Ethyl}}$, CH_2), 43.83 (s, 1C, $N_{3\text{-Ethyl}}$,

CH_2), 32.73 (d, $^1J_{Rh,C}$ = 49.7 Hz, 2C, CH_2-COD, CH_2), 29.11 (d, $^1J_{Rh,C}$ = 42.1 Hz, 2C, CH_2-COD, CH_2), 16.04 (s, 1C, N_3-Ethyl, CH_3), 16.01 (s, 1C, N_1-Ethyl, CH_3); ^{19}F NMR {^{1}H} (471 MHz, $CDCl_3$): δ (ppm) = -112.50 (tt, $^3J_{F,H}$ = 8.5 Hz, $^4J_{F,H}$ = 5.3 Hz); MS (ESI^+): m/z: 508.9 $[M]^+$; elemental analysis for $C_{21}H_{27}BrFN_2Rh$ (%calc./%found): C (49.53/49.94), H (5.34/5.46), N (5.50 / 5.35).

(Iodido)(cycloocta-1,5-dien)(1,3-diethyl-4-(4'-fluorphenylimidazol-2-yliden)) rhodium(I) **Rh-6**
Rh-4 (0.05 g) was dissolved in acetone (2.0 mL) in a microwave tube with a stirring core containing 2.0 eq. potassium iodide (0.036 g) and stirred for 30 min at 20 watts, 11 psi and 56 °C. The mixture is then transferred to a round bottom flask and the solvent removed under vacuo. The resulting substance is purified by column chromatography on silica gel (DCM). The isolated solid is dried in the drying gun in vacuum at 40 °C. Yield 92 % yellow crystalline solid. ^{1}H NMR (500 MHz, $CDCl_3$): δ (ppm) = 7.35-7.31 (m, 2H, $C_{2',6'}$, CH), 7.15-7.10 (m, 2H, $C_{3',5'}$, CH), 6.82 (s, 1H, C_5, CH), 5.26-5.20 (m, 2H, cis-CH-COD, CH), 4.82 (dq, $^2J_{H,H}$ = 13.5 Hz, $^3J_{H,H}$ = 7.4 Hz, 1H, $N_{3\text{-Ethyl}}$, CH_2), 4.65 (dq, $^2J_{H,H}$ = 13.4 Hz, $^3J_{H,H}$ = 7.4 Hz, 1H, $N_{1\text{-Ethyl}}$), 4.51 – 4.41 (m, 2H, 1H, $N_{1\text{-Ethyl}}$, + 1H, $N_{3\text{-Ethyl}}$, CH_2), 3.51 (m, 2H, trans-CH-COD, CH), 2.41-2.29 (m, 4H, CH_2-COD, $CH_{2,equatorial}$), 2.03-1.92 (m, 2H, CH_2-COD, $CH_{2,\ axial,cis}$), 1.86-1.77 (m, 2H, CH_2-COD, $CH_{2,\ axial,trans}$), 1.53 (t, $^3J_{H,H}$ = 7.4 Hz, 3H, $N_{1\text{-Ethyl}}$, CH_3), 1.19 (t, $^3J_{H,H}$ = 7.2 Hz, 3H, $N_{3\text{-Ethyl}}$, CH_3); ^{13}C NMR (126 MHz, $CDCl_3$): δ (ppm) = 182.18 (d, $^1J_{Rh,C}$ = 48.9 Hz, 1C, C_2, C-Rh), 162.91 (d, $^1J_{C,F}$ = 249.3 Hz, 1C, $C_{4'}$, C-F), 133.82 (s, 1C, C_4, C), 130.59 (d, $^3J_{C,F}$ = 8.3 Hz, 2C, $C_{2',6'}$, CH), 125.25 (d, $^4J_{C,F}$ = 3.5 Hz, 1C, $C_{1'}$, C), 118.48 (s, 1C, C_5, CH), 115.94 (d, $^2J_{C,F}$ = 21.8 Hz, 2C, $C_{3',5'}$, CH), 96.27 (m, 2C, cis-CH-COD, CH), 71.68 (dd, $^1J_{Rh,C}$ = 17.6 Hz, $^2J_{Rh,C}$ = 14.1 Hz, 2C, trans-CH-COD, CH), 45.73 (s, 1C, $N_{1\text{-Ethyl}}$, CH_2), 43.82 (s, 1C, $N_{3\text{-Ethyl}}$, CH_2), 32.33 (d, $^2J_{Rh,C}$ = 57.0 Hz, 2C, CH_2-COD, CH_2), 29.57 (d, $^2J_{Rh,C}$ = 48.6 Hz, 2C, CH_2-COD, CH_2), 15.60 (s, 1C, N_3-Ethyl, CH_3), 15.50 (s, 1C, N_1-Ethyl, CH_3); ^{19}F NMR (471 MHz, $CDCl_3$): δ (ppm) = -112.52 (tt, $^3J_{F,H}$ = 8.5 Hz, $^4J_{F,H}$ = 5.3 Hz); MS (ESI): m/z: 555.9 $[M]^+$; elemental analysis for $C_{21}H_{27}FIN_2Rh$ (%calc./%found): C (45.34 / 45.91), H (4.89 / 4.95), N (5.04 / 4.87).

(S-Thiocyanato)(cycloocta-1,5-dien)(1,3-diethyl-4-(4'-fluorphenylimidazol-2-yliden)) **Rh-7**
1,3-Diethyl-(4-(4'-Fluorphenyl)-1H-imidazol-2-ium) hexafluorophosphat (0.05 g) were dissolved in a microwave tube with 0.5 eq. methoxy(cycloocta-1,5-diene)rhodium dimer (0.033 g) and 2 eq. potassium thiocyanate (0.027 g) in 1.0 mL DCM and 2.0 mL ACN. The mixture was stirred at 20 watts, 11 psi and 50 °C for 1 h. The solution was transfer into a round-bottom flask and the solvent is removed under vacuo. The residue is taken up in DCM and filtered through a syringe filter (0.2 µm). The preparation is purified by column chromatography on silica gel (DCM/MeOH: [%] 100/0 → 100/0.5). The isolated solid is dried in the drying under vacuum at 40 °C. Yield 60 % yellow crystalline solid. ^{1}H NMR (500 MHz, $CDCl_3$): δ (ppm) =

7.36-7.31 (m, 2H, $C_{2',6'}$, CH), 7.15-7.10 (m, 2H, $C_{3',5'}$, CH), 6.87-6.83 (m, 1H, C_5, CH), 4.82-4.61 (m, 4H, 2H, cis-CH-COD, CH + 2H, 1H, $N_{3\text{-Ethyl}}$ + 1H, $N_{1\text{-Ethyl}}$, CH_2), 4.45 (dq, $^2J_{H,H}$ = 14.6 Hz, $^3J_{H,H}$ = 7.3 Hz, 2H, 1H, $N_{1\text{-Ethyl}}$ + 1H, $N_{3\text{-Ethyl}}$, CH_2), 3.68 (s, 2H, trans-CH-COD, CH), 2.49-2.35 (m, 4H, CH_2-COD, $CH_{2,\text{äquatorial}}$), 2.10-2.02 (m, 4H, CH_2-COD, $CH_{2,\text{ axial}}$), 1.56 (t, $^3J_{H,H}$ = 7.4 Hz, 3H, $N_{1\text{-Ethyl}}$, CH_3), 1.26 – 1.18 (m, 3H, $N_{3\text{-Ethyl}}$, CH_3); ^{13}C NMR (126 MHz, $CDCl_3$); δ (ppm) = 180.20 (d, $^1J_{Rh,C}$ = 60.9 Hz, 1C, C_2, C-Rh), 163.09 (d, $^1J_{C,F}$ = 249.6 Hz, 1C, $C_{4'}$, C-F), 134.59 (s, 1C, C_4, C), 130.97 (d, $^3J_{C,F}$ = 8.2 Hz, 2C, $C_{2',6'}$, CH), 124.89 (d, $^4J_{C,F}$ = 3.2 Hz, 1C, $C_{1'}$, C), 119.27 (s, 1C, C_5, CH), 115.96 (d, $^2J_{C,F}$ = 21.8 Hz, 2C, $C_{3',5'}$, CH), 96.30 (br. s, 2C, cis-CH-COD, CH), 74.73 (br. s, 2C, trans-CH-COD, CH), 45.87 (s, 1C, $N_{1\text{-Ethyl}}$, CH_2), 43.90 (s, 1C, $N_{3\text{-Ethyl}}$, CH_2), 32.54 (d, $^2J_{Rh,C}$ = 36.6 Hz, 2C, CH_2-COD, CH_2), 29.34 (d, $^2J_{Rh,C}$ = 30.8 Hz, 2C, CH_2-COD, CH_2), 15.99 (s, 1C, $N_{3\text{-Ethyl}}$, CH_3) 15.87 (s, 1C, $N_{1\text{-Ethyl}}$, CH_3); ^{19}F NMR (471 MHz, $CDCl_3$): δ (ppm) = -73.91 (d, $^1J_{F,P}$ = 712.2 Hz), -112.15 (s,); IR (KBr): 2103, 2080 cm^{-1} ($S=C=N^-$, N=C=S); MS (ESI^+): m/z: 487.0 $[M]^+$; elemental analysis for $C_{22}H_{27}FN_3RhS$ (%calc./%found): C (54.21 / 54.34), H (5.58 / 5.56), N (8.62 / 8.16).

(Chlorido)(cycloocta-1,5-dien)(1,3-dimethyl-4-(phenylimidazol-2-yliden))rhodium(I) **Rh-8**

1,3-Dimethyl-(4-phenyl-1H-imidazol-2-ium)iodid (0.1 g 0.34 mmol) was treated with 0.5 equivalents of Ag_2O (0.039 g, 0.17 mmol) for 4 h in 10 mL of dichloromethane under vigorous stirring at room temperature in the dark. After this period chlorido(cycloocta-1,5-dien)rhodium)dimer (0.082 g, 0.17 mmol) was added and the reaction was stirred 18 h. The obtained suspension was filtered over celite and the solvent was evaporated under reduced pressure to give an oil. The resulting oil is raised on silica gel and purified by column chromatography on silica gel (EtOAc/n-hexane: 1/1). The isolated solid is dried in the vacuum drying gun at 40 °C. Yield: 66% yellow crystalline solid. ^1H NMR (600 MHz, $CDCl_3$): δ (ppm) = 7.44-7.38 (m, 3H, $C_{3',4',5'}$, CH), 7.32-7.29 (m, 2H, $C_{2',6'}$, CH), 6.79 (s, 1H, C_5, CH), 5.08-5.02 (m, 2H, cis-CH-COD, CH), 4.12 (s, 3H, $N_{1\text{-Methyl}}$, CH_3), 4.06 (s, 3H, $N_{3\text{-Methyl}}$, CH_3), 3.40-3.35 (m, 2H, trans-CH-COD, CH), 2.52-2.35 (m, 4H, CH_2-COD, $CH_{2,\text{equatorial}}$), 2.01-1.93 (m, 4H, CH_2-COD, $CH_{2,\text{axial}}$); ^{13}C NMR (151 MHz, $CDCl_3$): δ (ppm) = 183.58 (d, $^1J_{Rh,C}$ = 51.1 Hz, 1C, C_2, C-Rh), 135.43 (s, 1C, C_4, C), 128.82-128.75 (m, 4C, $C_{2',3',5',6'}$, CH), 128.69 (s, 1C, $C_{4'}$, CH), 128.41 (s, 1C, $C_{1'}$, C), 119.83 (s, 1C, C_5, CH), 98.64 (dd, $^1J_{Rh,C}$ = 8.5 Hz, $^2J_{Rh,C}$ = 6.9 Hz, 2C, cis-CH COD, CH), 67.89 (dd, $^1J_{Rh,C}$ = 14.7 Hz, $^2J_{Rh,C}$ = 8.7 Hz, 2C, trans-CH COD, CH), 37.83 (s, 1C, $N_{1\text{-Methyl}}$, CH_3), 36.19 (s, 1C, $N_{3\text{-Methyl}}$, CH_3), 32.99 (d, $^2J_{Rh,C}$ = 9.1 Hz, 2C, CH_2-COD, CH_2), 28.91 (d, $^2J_{Rh,C}$ = 11.5 Hz, 2C, CH_2-COD, CH_2); MS (ESI^+): m/z: 418.0 $[M]^+$; elemental analysis for $C_{19}H_{24}ClN_2Rh$ (%calc./%found): C (54.40 / 54.45), H (5.78 / 5.64), N (6.69 / 6.65).

(Bromido)(cycloocta-1,5-dien)(1,3-dimethyl-4-(phenylimidazol-2-yliden))rhodium(I) **Rh-9**

1,3-Dimethyl-(4-phenyl-1H-imidazol-2-ium)iodid (0.1 g 0.34 mmol) was treated with 0.5 equivalents of Ag_2O (0.039 g, 0.17 mmol) for 4 h in 10 mL of dichloromethane under vigorous stirring at room temperature in the dark. After this period bromido(cycloocta-1,5-dien)rhodium)dimer (0.098 g, 0.17 mmol) was added and the reaction was stirred 18 h. The obtained suspension was filtered over celite and the solvent was evaporated under reduced pressure to give an oil. The resulting oil is raised on silica gel and purified by column chromatography on silica gel (EtOAc/n-hexane: 1/1). The isolated solid is dried in the vacuum drying gun at 40 °C. Yield: 78% yellow crystalline solid. ^{1}H NMR (600 MHz, $CDCl_3$): δ (ppm) = 7.44-7.38 (m, 3H, $C_{3',4',5'}$, CH), 7.33-7.30 (m, 2H, $C_{2',6'}$, CH), 6.81 (s, 1H, C_5, CH), 5.14-5.09 (m, 2H, cis-CH-COD, CH), 4.08 (s, 3H, $N_{1\text{-Methyl}}$, CH_3), 4.03 (s, 3H, $N_{3\text{-Methyl}}$, CH_3), 3.45-3.40 (m, 2H, trans-CH-COD, CH), 2.45-2.33 (m, 4H, CH_2-COD, $CH_{2,\text{equatorial}}$), 2.02-1.94 (m, 2H, CH_2-COD, $CH_{2,\text{axial,cis}}$), 1.94-1.87 (m, 2H, CH_2-COD, $CH_{2,\text{axial,trans}}$); ^{13}C NMR (151 MHz, $CDCl_3$): δ (ppm) = 183.48 (d, $^{1}J_{Rh,C}$ = 50.1 Hz, 1C, C_2, C-Rh), 135.49 (s, 1C, C_4, C), 128.81-128.73 (m, 4C, $C_{2',3',5',6'}$, CH), 128.68 (s, 1C, $C_{4'}$, CH), 128.41 (s, 1C, $C_{1'}$, C), 119.92 (s, 1C, C_5, CH), 98.02 (dd, $^{1}J_{Rh,C}$ = 8.5 Hz, $^{2}J_{Rh,C}$ 6.7 Hz, 2C, cis-CH-COD, CH), 68.85 (dd, $^{1}J_{Rh,C}$ = 14.6 Hz, $^{2}J_{Rh,C}$ = 10.3 Hz, 2C, trans-CH-COD, CH), 37.85 (s, 1C, $N_{1\text{-Methyl}}$, CH_3), 36.20 (s, 1C, $N_{3\text{-Methyl}}$, CH_3), 32.76 (d, $^{2}J_{Rh,C}$ = 12.0 Hz, 2C, CH_2-COD, CH_2), 29.13 (d, $^{2}J_{Rh,C}$ = 14.1 Hz, 2C, CH_2-COD, CH_2); MS (ESI^+): m/z: 463.9 $[M]^+$; elemental analysis for $C_{19}H_{24}BrN_2Rh$ (%calc. / %found): C (49.27 / 49.62), H (5.22 / 5.13), N (6.05 / 5.83.

(Chlorido)(cycloocta-1,5-dien)(1,3-dimethyl-4-(4'-fluorphenylimidazol-2-yliden)) **Rh-10**

1,3-Dimethyl-(4-(4'-Fluorphenyl)-1H-imidazol-2-ium) iodide (0.1 g 0.30 mmol) was treated with 0.5 equivalents of Ag_2O (0.036 g, 0.15 mmol) for 4 h in 10 mL of dichloromethane under vigorous stirring at room temperature in the dark. After this period Chlorido(cycloocta-1,5-dien)rhodium)dimer (0.078 g, 0.15 mmol) was added and the reaction was stirred 18 h. The obtained suspension was filtered over celite and the solvent was evaporated under reduced pressure to give an oil. The resulting oil is raised on silica gel and purified by column chromatography on silica gel (EtOAc/n-hexane: 1/1). The isolated solid is dried in the vacuum drying gun at 40 °C. Yield: 80% yellow crystalline solid. ^{1}H NMR (600 MHz, $CDCl_3$) δ (ppm) = 7.30-7.27 (m, 2H, $C_{2',6'}$, CH), 7.15-7.10 (m, 2H, $C_{3',5'}$, CH), 6.78 (s, 1H, C_5, CH), 5.07-5.02 (m, 2H, cis-CH-COD, CH), 4.11 (s, 3H, $N_{1\text{-Methyl}}$, CH_3), 4.03 (s, 3H, $N_{3\text{-Methyl}}$, CH_3), 3.39-3.34 (m, 2H, trans-CH-COD, CH), 2.52-2.36 (m, 4H, CH_2-COD, $CH_{2,\text{äquatorial}}$), 2.02-1.93 (m, 4H, CH_2-COD, $CH_{2,\text{axial}}$); ^{13}C NMR (151 MHz, $CDCl_3$) δ (ppm) = 183.80 (d, $^{1}J_{Rh,C}$ = 51.1 Hz, 1C, C_2, C-Rh), 162.96 (d, $^{1}J_{C,F}$ = 249.5 Hz, 1C, $C_{4'}$, C), 134.37 (s, 1C, C_4, CH), 130.79 (d, $^{3}J_{C,F}$ = 8.3 Hz, 2C, $C_{2',6'}$, CH), 124.46 (d, $^{4}J_{C,F}$ = 3.4 Hz, 1C, $C_{1'}$, C), 119.93 (s, 1C, C_5, CH), 115.95 (d, $^{2}J_{C,F}$ = 21.8 Hz, 2C, $C_{3',5'}$, CH), 98.75 (dd, $^{1}J_{Rh,C}$ = 8.4 Hz, $^{2}J_{Rh,C}$ = 6.9 Hz, 2C, cis-CH-COD, CH), 67.90 (dd, $^{1}J_{Rh,C}$ = 14.6 Hz, $^{2}J_{Rh,C}$ = 9.5 Hz, 2C, trans-CH-COD, CH), 37.85 (s, 1C, $N_{1\text{-Methyl}}$, CH_3), 36.08 (s,

1C, $N_{3\text{-Methyl}}$, CH_3), 32.98 (d, $^2J_{Rh,C}$ = 11.3 Hz, 2C, CH_2-COD, CH_2), 28.90 (d, $^2J_{Rh,C}$ = 13.1 Hz, 2C, CH_2-COD, CH_2); ^{19}F NMR (471 MHz, $CDCl_3$) δ (ppm) = -112.40 (tt, $^3J_{F,H}$ = 8.4 Hz, $^4J_{F,H}$ = 5.3 Hz); MS (ESI$^+$): m/z: 436.0 [M]$^+$; elemental analysis for $C_{19}H_{23}ClFN_2Rh$ (%calc. / %found): C (52.25 / 51.93), H (5.31 / 5.29), N (6.41 / 6.19).

(Bromido)(cycloocta-1,5-dien)(1,3-dimethyl-4-(4'-fluorphenylimidazol-2-ylidene))rhodium(I) **Rh-11**

1,3-Dimethyl-(4-(4'-Fluorphenyl)-1H-imidazol-2-ium)iodid (0.055 g 0.17 mmol) was treated with 0.5 equivalents of Ag_2O (0.02 g, 0.086 mmol) for 4 h in 10 mL of dichloromethane under vigorous stirring at room temperature in the dark. After this period bromido(cycloocta-1,5-dien)rhodium)dimer (0.078 g, 0.086 mmol) was added and the reaction was stirred 18 h. The obtained suspension was filtered over celite and the solvent was evaporated under reduced pressure to give an oil. The resulting oil is raised on silica gel and purified by column chromatography on silica gel (EtOAc/n-hexane: 1/1). The isolated solid is dried in the vacuum drying gun at 40 °C. Yield: 80% yellow crystalline solid. ^{1}H NMR (500 MHz, $CDCl_3$) δ (ppm) = 7.31-7.27 (m, 2H, $C_{2',6'}$, CH), 7.15-7.10 (m, 2H, $C_{3',5'}$, CH), 6.79 (s, 1H, C_5, CH), 5.15-5.08 (m, 2H, *cis*-CH-COD, CH), 4.08 (s, 3H, $N_{1\text{-Methyl}}$, CH_3), 4.00 (s, 3H, $N_{3\text{-Methyl}}$, CH_3), 3.45-3.39 (m, 2H, *trans*-CH-COD, CH), 2.52-2.34 (m, 4H, CH_2-COD, $CH_{2,equatorial}$), 2.04-1.86 (m, 4H, CH_2-COD, $CH_{2,axial}$); ^{13}C NMR (126 MHz, $CDCl_3$) δ (ppm) = 183.71 (d, $^1J_{Rh,C}$ = 50.1 Hz, 1C, C_2, C-Rh), 162.95 (d, $^1J_{C,F}$ = 249.5 Hz, 1C, $C_{4'}$, C-F), 134.44 (s, 1C, C_4, CH), 130.78 (d, $^3J_{C,F}$ = 8.3 Hz, 2C, $C_{2',6'}$, CH), 124.47 (d, $^4J_{C,F}$ = 3.4 Hz, 1C, $C_{1'}$, C), 120.02 (s, 1C, C_5, CH), 115.94 (d, $^2J_{C,F}$ = 21.7 Hz, 2C, $C_{3',5'}$, CH), 98.13 (dd, $^2J_{Rh,C}$ = 6.6 Hz, 2C, cis-CH-COD, CH), 68.86 (dd, $^1J_{Rh,C}$ = 14.6 Hz, $^2J_{Rh,C}$ = 8.0 Hz, 2C, trans-CH-COD, CH), 37.87 (s, 1C, $N_{1\text{-Methyl}}$, CH_3), 36.10 (s, 1C, $N_{3\text{-Methyl}}$, CH_3), 32.76 (d, $^2J_{Rh,C}$ = 10.8 Hz, 2C, CH_2-COD, CH_2), 29.12 (d, $^2J_{Rh,C}$ = 12.1 Hz, 2C, CH_2-COD, CH_2); ^{19}F NMR (471 MHz, $CDCl_3$) δ (ppm) = -112.42 (tt, $^3J_{F,H}$ = 8.5 Hz, $^4J_{F,H}$ = 5.2 Hz); MS (ESI$^+$): m/z: 479.9 [M]$^+$; elemental analysis for $C_{19}H_{23}BrFN_2Rh$ (%calc. / %found): C (47.42 / 47.79), H (4.82 / 4.72), N (5.82 / 5.53).

4) Synthesis of iron compound Fe12

General Fe-12: (Same as Ru-17) NMR were run on Bruker Avance III 600 spectrometer; Mass spectra were recorded using EI method on a Finnigan MAT 95 mass spectrometer and ESI on a Synapt G2-Si mass spectrometer (Waters); IR spectra were recorded on an FTIR Nexus Nicolet apparatus; Microanalyses were performed by the Analytical Services of the Polish Academy of the Sciences (Łódź).

1,1'-bis[(*E*)-(3-(1-thyminyl)-prop-1-ene)]ferrocene **Fe12**

Ytterbium(III) trifluoromethanesulfonate (10 mg, 0.02 mmol) was added to solution of 1,1'-bis(3-(1-thyminyl)-propan-1-ol)ferrocene (100 mg, 0.18 mmol) in 1,3-propanediol (10 mL) at ambient temperature. The reaction mixture was stirred at 100 °C for 10 h. Then, the reaction was cool to ambient temperature, poured into water, and the obtained mixture was extracted with chloroform. The organic layer was separated, dried over anhydrous $MgSO_4$, filtered and all volatiles were evaporated under reduced pressure. The remaining material was subjected to column chromatography on silica gel with dichloromethane / methanol (50 : 1 v/v) as eluent. Crystallization from a dichloromethane/*n*-pentane gave analytically pure compound. Yield: 25 % (25 mg), orange solid. ^{1}H NMR (600 MHz, DMSO-d_6): δ (ppm) = 11.25 (s, 2H, NH), 7.49 (d, 2H, J = 0.6 Hz, H6 thymine), 6.23 (d, 2H, J = 16.2 Hz, vinyl), 5.79 (dt, 2H, J = 16.2 Hz, 6.0 Hz, vinyl), 4.28 (pt, 4H, J = 1.8 Hz, C_5H_4), 4.25 (d, 4H, J = 6.0 Hz, CH_2), 4.17 (pt, 4H, J = 1.8 Hz, C_5H_4), 1.77 (d, 6H, J = 0.6 Hz, CH_3 thymine). ^{13}C NMR (150 MHz, DMSO-d_6): δ (ppm) = 164.4, 150.8, 141.0, 130.6, 121.1, 108.9, 82.4, 70.0, 68.2, 48.9, 12.0; MS (EI, 70eV): m/z = 514 $[M]^+$. FTIR (KBr ν [cm^{-1}]): 2914, 1699, 1666, 1463, 1436, 1348, 1222; elemental analysis for $C_{26}H_{26}N_2O_4Fe$ (%calc. / %found): C (60.71 / 60.88), H (5.10 / 5.15), N (10.89 / 10.87).

5) Synthesis of silver complex Ag-3

General Ag-3:

NMR spectra were recorded on a Varian NMR 400 (^{1}H 399.5 MHz; ^{13}C{^{1}H} 100.5 MHz or 500 (^{1}H 499.9 MHz; ^{13}C{^{1}H} 125.7 MHz) and chemical shifts are reported in ppm. Spectra are referenced to the corresponding protic solvent (^{1}H) or signals of the solvent (^{13}C). LC-MS purity analyses were undertaken using a 5 µm C18 110 Å column. Percentage purities were performed using a 30 min method in water/acetonitrile with 0.1% formic acid (5 min at 5%, 5−95% over 20 min, 5 min at 95%) with the UV set to 254 nm. High-resolution mass spectrometry was carried out at the University of Sussex.

Ag-3 was obtained by a synthesis procedure described by de Fremont et al. in *Organometallics* 2005, 24, 6301-6309.
White solid made in 80% yield. ^{1}H NMR (600 MHz, Chloroform-d) δ 7.01 (s, 2H), 4.67 (sep, J = 6.8 Hz, 2H), 1.44 (d, J = 6.8 Hz, 12H). ^{13}C NMR (151 MHz, Chloroform-d) δ 117.4, 54.4, 23.9 (carbonic carbon not detected). HRMS cald for $C_{18}H_{32}AgN_4^+$ 411.1672; found 411.1655.

6) Tables S1 to S3 (Results of the inhibitor assays)

Table S1: Results of the S/ACE2 ELISA assay (n.d.: not determined)

compound (20 µM)	inhibition (% ± SD)	compound (20 µM)	inhibition (% ± SD)	compound (20 µM)	inhibition (% ± SD)
**CQ**	39 ± 1	**Ru-1**	7 ± 6	**Hg-1**	7 ± 0
**Au-1**	20 ± 9	**Ru-2**	0 ± 12	**Ti-1**	69 ± 2
**Au-2**	18 ± 1	**Ru-3**	20 ± 12	**Ti-2**	0 ± 7
**Au-3**	24 ± 2	**Ru-4**	0 ± 10	**Pt-1**	25 ± 6
**Au-4**	19 ± 1	**Ru-5**	0 ± 7	**Pt-2**	18 ± 0
**Au-5**	45 ± 5	**Ru-6**	22 ± 7	**Pt-3**	2 ± 3
**Au-6**	16 ± 2	**Ru-7**	0 ± 16	**Pt-4**	7 ± 0
**Au-7**	18 ± 4	**Ru-8**	0 ± 17	**Pd-1**	13 ± 8
**Au-8**	13 ± 3	**Ru-9**	2 ± 3	**Pd-2**	21 ± 4
**Au-9**	21 ± 3	**Ru-10**	8 ± 10	**Fe-1**	2 ± 26
**Au-10**	21 ± 0	**Ru-11**	10 ± 10	**Fe-2**	21 ± 0
**Au-11**	-1 ± 16	**Ru-12**	6 ± 2	**Fe-3**	9 ± 6
**Au-12**	19 ± 8	**Ru-13**	7 ± 4	**Fe-4**	17 ± 3
**Au-13**	10 ± 1	**Ru-14**	25 ± 9	**Fe-5**	22 ± 10
**Au-14**	17 ± 4	**Ru-15**	25 ± 9	**Fe-6**	5 ± 0
**Au-15**	11 ± 13	**Ru-16**	31 ± 2	**Fe-7**	20 ± 5
**Au-16**	10 ± 2	**Ru-17**	26 ± 8	**Fe-8**	n.d.
**Au-17**	9 ± 4	**Mn-1**	21 ± 7	**Fe-9**	25 ± 1
**Au-18**	10 ± 5	**Mn-2**	23 ± 5	**Fe-10**	22 ± 4
**Au-19**	16 ± 4	**Re-1**	19 ± 10	**Fe-11**	0 ± 22
**Au-20**	-10 ± 24	**Re-2**	24 ± 6	**Fe-12**	28 ± 5
**Au-21**	22 ± 4	**Rh-1**	0 ± 4	**Fe-13**	25 ± 4
**Au-22**	23 ± 0	**Rh-2**	1 ± 5	**POM-1**	52 ± 7
**Au-23**	23 ± 3	**Rh-3**	0 ± 9	**POM-2**	50 ± 9
**Au-24**	18 ± 1	**Rh-4**	6 ± 1	**POM-3**	68 ± 18
**Au-25**	0 ± 29	**Rh-5**	5 ± 0	**POM-4**	82 ± 10
**Au-26**	0 ± 27	**Rh-6**	22 ± 8	**POM-5**	7 ± 3
**Au-27**	9 ± 13	**Rh-7**	16 ± 8	**POM-6**	91 ± 12
**Au-28**	0 ± 9	**Rh-8**	12 ± 3	**POM-7**	98 ± 1
**Au-29**	8 ± 10	**Rh-9**	17 ± 1	**POM-8**	9 ± 7
**Au-30**	15 ± 8	**Rh-10**	14 ± 0	**POM-9**	7 ± 4
**Au-31**	16 ± 1	**Rh-11**	14 ± 3	**POM-10**	6 ± 3
**Au-32**	5 ± 13	**Ag-1**	6 ± 0	**POM-11**	75 ± 3
**Au-33**	10 ± 10	**Ag-2**	4 ± 0		
**Au-34**	15 ± 10	**Ag-3**	27 ± 4		
**Au-35**	0 ± 8				
**Au-36**	0 ± 5				

Table S2: Results of the FRET assay with SARS-CoV PL^{pro}

compound (10 µM)	inhibition (% ± SD)	compound (10 µM)	inhibition (% ± SD)	compound (10 µM)	inhibition (% ± SD)
**DS**	87 ± 3	**Ru-1**	25 ± 6	**Hg-1**	-63 ± 41
**Au-1**	58 ± 2	**Ru-2**	20 ± 10	**Ti-1**	6 ± 21
**Au-2**	91 ± 4	**Ru-3**	18 ± 30	**Ti-2**	-7 ± 3
**Au-3**	95 ± 2	**Ru-4**	8 ± 2	**Pt-1**	34 ± 7
**Au-4**	50 ± 8	**Ru-5**	10 ± 4	**Pt-2**	75 ± 8
**Au-5**	89 ± 1	**Ru-6**	53 ± 2	**Pt-3**	2 ± 1
**Au-6**	0 ± 9	**Ru-7**	52 ± 2	**Pt-4**	-1 ± 3
**Au-7**	92 ± 2	**Ru-8**	9 ± 1	**Pd-1**	40 ± 22
**Au-8**	100 ± 0	**Ru-9**	12 ± 1	**Pd-2**	17 ± 2
**Au-9**	100 ± 0	**Ru-10**	16 ± 3	**Fe-1**	10 ± 5
**Au-10**	100 ± 0	**Ru-11**	21 ± 10	**Fe-2**	1 ± 1
**Au-11**	5 ± 5	**Ru-12**	46 ± 29	**Fe-3**	-5 ± 10
**Au-12**	93 ± 5	**Ru-13**	44 ± 21	**Fe-4**	-14 ± 24
**Au-13**	100 ± 0	**Ru-14**	6 ± 2	**Fe-5**	9 ± 6
**Au-14**	51 ± 1	**Ru-15**	10 ± 0	**Fe-6**	19 ± 1
**Au-15**	100 ± 0	**Ru-16**	10 ± 2	**Fe-7**	19 ± 9
**Au-16**	57 ± 5	**Ru-17**	3 ± 0	**Fe-8**	9 ± 9
**Au-17**	-3 ± 23	**Mn-1**	1 ± 6	**Fe-9**	12 ± 0
**Au-18**	85 ± 1	**Mn-2**	5 ± 7	**Fe-10**	8 ± 1
**Au-19**	94 ± 3	**Re-1**	2 ± 1	**Fe-11**	16 ± 7
**Au-20**	83 ± 2	**Re-2**	7 ± 2	**Fe-12**	-4 ± 9
**Au-21**	11 ± 5	**Rh-1**	67 ± 12	**Fe-13**	6 ± 4
**Au-22**	39 ± 4	**Rh-2**	67 ± 9	**POM-1**	100 ± 0
**Au-23**	25 ± 3	**Rh-3**	63 ± 9	**POM-2**	100 ± 0
**Au-24**	12 ± 6	**Rh-4**	72 ± 4	**POM-3**	63 ± 10
**Au-25**	21 ± 7	**Rh-5**	71 ± 5	**POM-4**	44 ± 9
**Au-26**	15 ± 4	**Rh-6**	70 ± 10	**POM-5**	49 ± 22
**Au-27**	22 ± 1	**Rh-7**	45 ± 1	**POM-6**	80 ± 3
**Au-28**	12 ± 11	**Rh-8**	93 ± 1	**POM-7**	92 ± 5
**Au-29**	24 ± 7	**Rh-9**	94 ± 3	**POM-8**	5 ± 5
**Au-30**	100 ± 0	**Rh-10**	90 ± 3	**POM-9**	13 ± 13
**Au-31**	100 ± 0	**Rh-11**	91 ± 1	**POM-10**	7 ± 7
**Au-32**	100 ± 0	**Ag-1**	100 ± 0	**POM-11**	100 ± 0
**Au-33**	78 ± 13	**Ag-2**	-7 ± 13		
**Au-34**	86 ± 18	**Ag-3**	-9 ± 21		
**Au-35**	85 ± 5				
**Au-36**	79 ± 1				

Table S3: Results of the FRET assay with SARS-CoV-2 PL^{pro}

compound (1.0 µM)	inhibition (% ± SD)	compound (1.0 µM)	inhibition (% ± SD)	compound (1.0 µM)	inhibition (% ± SD)
**DS**	73 ± 12	**Ru-1**	10 ± 7	**Hg-1**	9 ± 4
**Au-1**	51 ± 14	**Ru-2**	5 ± 11	**Ti-1**	-13 ± 10
**Au-2**	64 ± 1	**Ru-3**	3 ± 10	**Ti-2**	-3 ± 16
**Au-3**	55 ± 2	**Ru-4**	-7 ± 8	**Pt-1**	19 ± 3
**Au-4**	78 ± 8	**Ru-5**	-1 ± 9	**Pt-2**	-5 ± 15
**Au-5**	72 ± 5	**Ru-6**	3 ± 9	**Pt-3**	-19 ± 9
**Au-6**	-6 ± 7	**Ru-7**	2 ± 11	**Pt-4**	-3 ± 3
**Au-7**	31 ± 5	**Ru-8**	7 ± 7	**Pd-1**	-2 ± 12
**Au-8**	55 ± 8	**Ru-9**	10 ± 1	**Pd-2**	50 ± 20
**Au-9**	86 ± 5	**Ru-10**	-7 ± 7	**Fe-1**	-1 ± 21
**Au-10**	42 ± 2	**Ru-11**	5 ± 7	**Fe-2**	-4 ± 8
**Au-11**	14 ± 11	**Ru-12**	-7 ± 13	**Fe-3**	2 ± 6
**Au-12**	60 ± 4	**Ru-13**	-8 ± 18	**Fe-4**	0 ± 0
**Au-13**	65 ± 4	**Ru-14**	-4 ± 10	**Fe-5**	-4 ± 3
**Au-14**	23 ± 2	**Ru-15**	-3 ± 13	**Fe-6**	-2 ± 3
**Au-15**	48 ± 3	**Ru-16**	5 ± 13	**Fe-7**	-2 ± 8
**Au-16**	41 ± 17	**Ru-17**	-17 ± 5	**Fe-8**	-2 ± 8
**Au-17**	13 ± 24	**Mn-1**	-18 ± 6	**Fe-9**	2 ± 5
**Au-18**	11 ± 3	**Mn-2**	-4 ± 6	**Fe-10**	-1 ± 12
**Au-19**	2 ± 2	**Re-1**	-4 ± 7	**Fe-11**	-31 ± 12
**Au-20**	30 ± 0	**Re-2**	-10 ± 3	**Fe-12**	-8 ± 10
**Au-21**	10 ± 5	**Rh-1**	-4 ± 10	**Fe-13**	-4 ± 1
**Au-22**	7 ± 1	**Rh-2**	17 ± 1	**POM-1**	68 ± 11
**Au-23**	-10 ± 6	**Rh-3**	15 ± 1	**POM-2**	66 ± 4
**Au-24**	-17 ± 1	**Rh-4**	17 ± 2	**POM-3**	15 ± 7
**Au-25**	9 ± 1	**Rh-5**	19 ± 1	**POM-4**	7 ± 7
**Au-26**	12 ± 1	**Rh-6**	19 ± 2	**POM-5**	69 ± 1
**Au-27**	0 ± 0	**Rh-7**	6 ± 13	**POM-6**	58 ± 5
**Au-28**	-18 ± 2	**Rh-8**	35 ± 1	**POM-7**	67 ± 7
**Au-29**	4 ± 12	**Rh-9**	50 ± 16	**POM-8**	7 ± 3
**Au-30**	38 ± 8	**Rh-10**	45 ± 12	**POM-9**	7 ± 3
**Au-31**	73 ± 8	**Rh-11**	48 ± 15	**POM-10**	4 ± 4
**Au-32**	65 ± 0	**Ag-1**	72 ± 3	**POM-11**	85 ± 3
**Au-33**	99 ± 1	**Ag-2**	33 ± 16		
**Au-34**	98 ± 2	**Ag-3**	69 ± 8		
**Au-35**	58 ± 18				
**Au-36**	74 ± 9				

7) Toxicity against cells

Method

Caco-2 and Calu-3 cells were grown as almost confluent monolayers in 96 well plates. The complexes were dissolved as stock solutions in DMSO or water (**Au-2**, **Ag-1**, **Ag-2** and POMs) and were diluted with DMEM cell culture medium, which was supplemented with 10% fetal calf serum, to the indicated concentrations. The cell layers were incubated with the drug containing media for 24 h at 37°C / 5% CO_2 in an incubator. The cell viability was determined by crystal violet staining and cell viability was calculated as percentage of an untreated control. Results were obtained in three independent experiments.

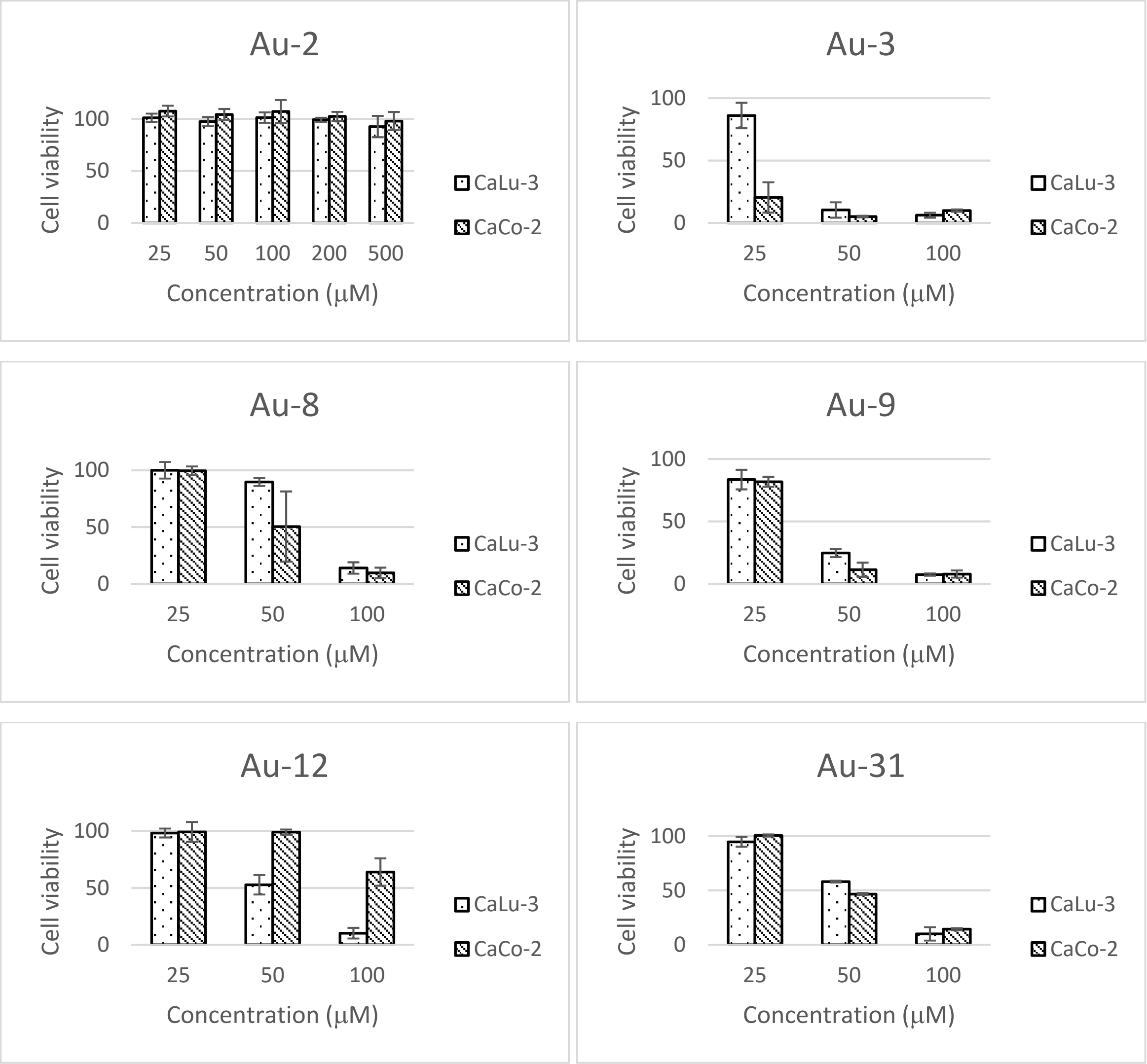

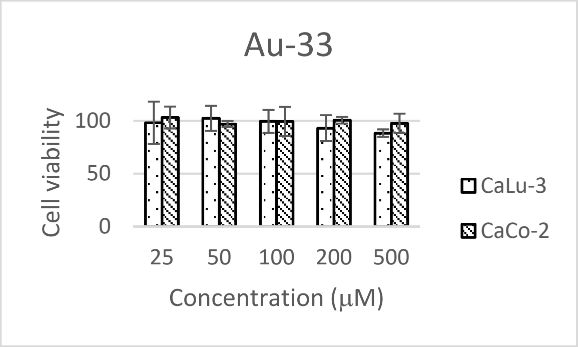
Au-33
Cell viability
100
50
0
25
50
100
200
500
Concentration (μM)
CaLu-3
CaCo-2

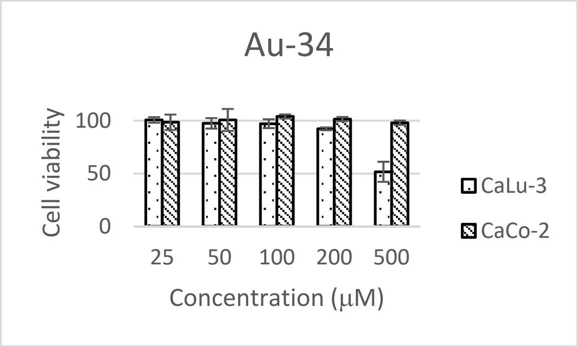
Au-34
Cell viability
100
50
0
25
50
100
200
500
Concentration (μM)
CaLu-3
CaCo-2

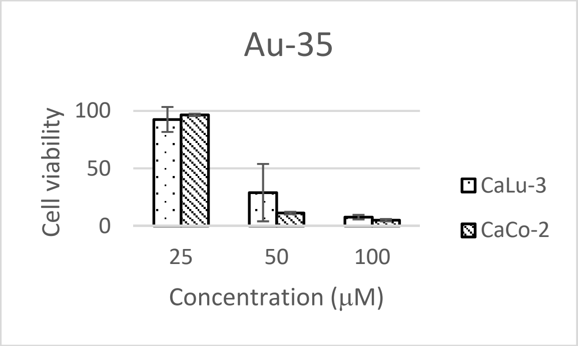
Au-35
Cell viability
100
50
0
25
50
100
Concentration (μM)
CaLu-3
CaCo-2

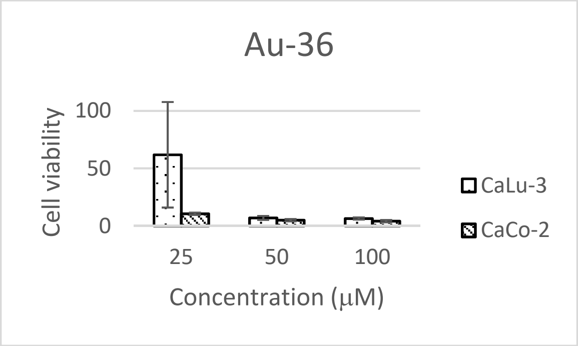
Au-36
Cell viability
100
50
0
25
50
100
Concentration (μM)
CaLu-3
CaCo-2

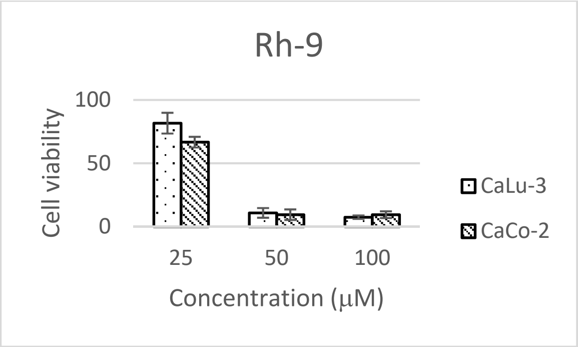
Rh-9
Cell viability
100
50
0
25
50
100
Concentration (μM)
CaLu-3
CaCo-2

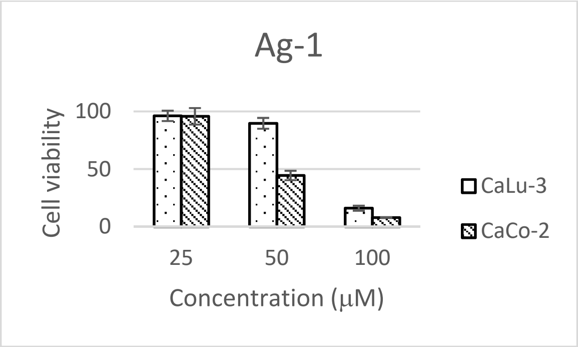
Ag-1
Cell viability
100
50
0
25
50
100
Concentration (μM)
CaLu-3
CaCo-2

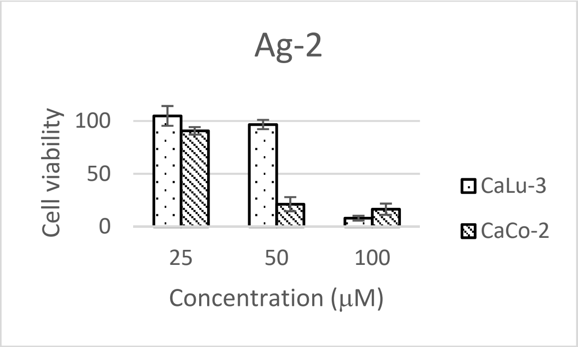
Ag-2
Cell viability
100
50
0
25
50
100
Concentration (μM)
CaLu-3
CaCo-2

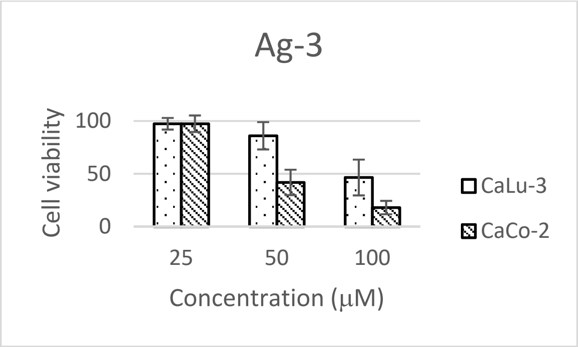
Ag-3
Cell viability
100
50
0
25
50
100
Concentration (μM)
CaLu-3
CaCo-2

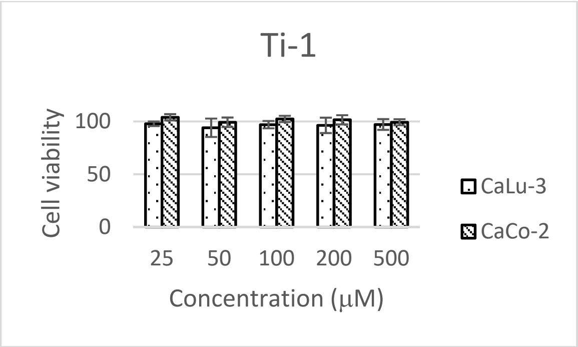
Ti-1
Cell viability
100
50
0
25
50
100
200
500
Concentration (μM)
CaLu-3
CaCo-2

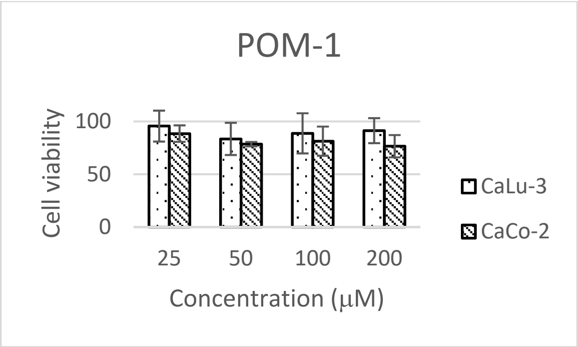
POM-1
Cell viability
100
50
0
25
50
100
200
Concentration (μM)
CaLu-3
CaCo-2

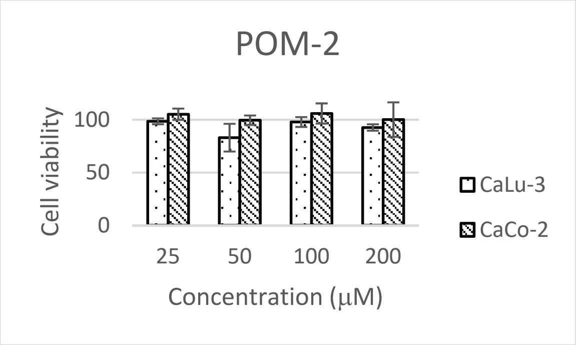
POM-2
Cell viability
100
50
0
25
50
100
200
Concentration (µM)
CaLu-3
CaCo-2

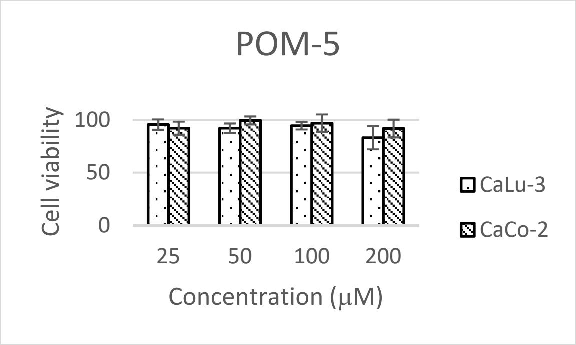
POM-5
Cell viability
100
50
0
25
50
100
200
Concentration (µM)
CaLu-3
CaCo-2

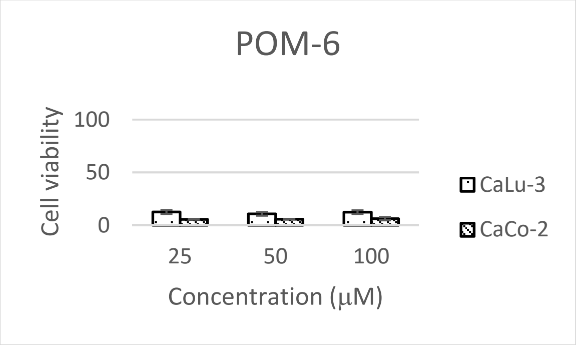
POM-6
Cell viability
100
50
0
25
50
100
Concentration (µM)
CaLu-3
CaCo-2

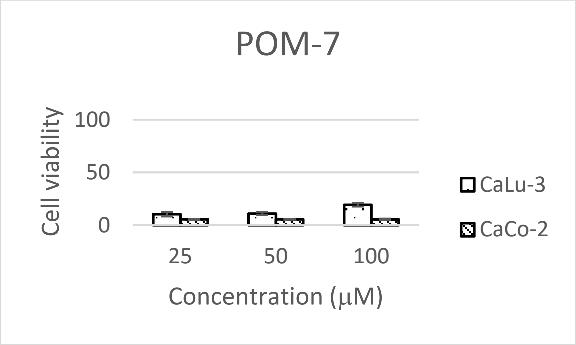
POM-7
Cell viability
100
50
0
25
50
100
Concentration (µM)
CaLu-3
CaCo-2

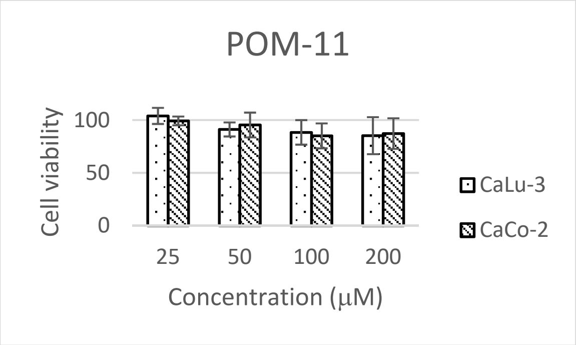
POM-11
Cell viability
100
50
0
25
50
100
200
Concentration (µM)
CaLu-3
CaCo-2
